# Physiological and Biochemical Responses of *Nitraria tangutorum* to Long-Term Saline Irrigation in an Arid Coal-Mining Restoration Area

**DOI:** 10.3390/ijms27177694

**Published:** 2026-08-28

**Authors:** Abdul Waheed, Xu Qiao, Haiyan Wang, Meiquan Li, Xinlong Li, Tongxin Wang, Aili Aishajiang, Hailiang Xu

**Affiliations:** 1 State Key Laboratory of Desert and Oasis, Xinjiang Institute of Ecology and Geography, Chinese Academy of Sciences, Urumqi 830011, China; drwaheed@ms.xjb.ac.cn; 2 College of Resources and Environment, Yili Normal University, Yining 835000, China; 3 Institute of Resources and Ecology, Yili Normal University, Yining 835000, China; 4 Xinjiang Uygur Autonomous Region Environmental Engineering Evaluation Center, Urumqi 830011, China; haiyanwang_xje@163.com; 5 Xinjiang Zhongneng Electric Power Development Co., Ltd., Hami 839000, China; 6 School of Geographical Sciences and Tourism, Xinjiang Normal University, Urumqi 830010, China

**Keywords:** *Nitraria tangutorum*, saline irrigation, physiological regulation, antioxidant defense, ascorbate–glutathione cycle, ecological restoration, arid coal mining areas

## Abstract

Saline irrigation is a major constraint on vegetation establishment in arid coal-mining landscapes. *Nitraria tangutorum* Bobrov is a xerohalophytic shrub established in the Dananhu mine-restoration area of Hami, Xinjiang, but its physiological responses to long-term saline irrigation remain insufficiently characterized. In this field study, approximately two-year-old plants were maintained for approximately two growing seasons under freshwater drip irrigation or irrigation water containing 8 or 12 g L^−1^ total dissolved solids. Unlike short-term controlled salinity assays that evaluate individual response pathways, the present field study integrates root and shoot responses across osmolyte accumulation, oxidative injury, redox regulation, nitrogen metabolism, phytohormone signaling, and lipid remodeling under long-term mixed-salt irrigation. Increasing salinity significantly reduced shoot total chlorophyll content, whereas shoot carotenoid content showed a nonsignificant numerical increase. Soluble sugars, proline, and soluble proteins increased, while total free amino acids declined. Superoxide anion (O_2_^−^), hydrogen peroxide (H_2_O_2_), and malondialdehyde (MDA) increased progressively, demonstrating oxidative injury. The protein concentrations of superoxide dismutase (SOD), catalase (CAT), peroxidase (POD), and ascorbate peroxidase (APX) increased, whereas glutathione peroxidase (GSH-PX) and glutathione reductase (GR) declined. Concurrent decreases in ascorbic acid/ascorbate (AsA) and reduced glutathione (GSH), together with increases in dehydroascorbic acid (DHA) and oxidized glutathione (GSSG), indicated progressive oxidation of the cellular redox environment. Nitrate reductase (NR) declined under salinity, while glutamine synthetase (GS) and glutamate synthase (GOGAT) increased at 8 g L^−1^ but decreased at 12 g L^−1^. Growth-associated hormones declined, whereas stress-associated hormones increased. Together, these responses reveal a dose-dependent transition from co-occurring biochemical adjustment and oxidative injury at 8 g L^−1^ to broader redox and metabolic disruption at 12 g L^−1^ under long-term field irrigation. These variables provide candidate indicators of downstream salinity response, but ion homeostasis, plant water status, growth, survival, and long-term performance must be evaluated before salt tolerance or irrigation thresholds can be established.

## 1. Introduction

Coal-mining disturbance can strongly degrade soil structure, hydrological conditions, and vegetation, and ecological recovery is particularly difficult in arid regions where low precipitation, high evaporative demand, poor substrate development, and salinity jointly restrict plant establishment [1,2,3]. These constraints are pronounced in the Hami mining region of Xinjiang, where freshwater available for ecological irrigation is limited and saline or mineralized water may therefore be used for vegetation restoration [4,5]. A previous field study in the Dananhu restoration area evaluated *Tamarix chinensis*, *Calligonum mongolicum, Haloxylon ammodendron*, and *Phragmites australis* under irrigation-water salinities of 8, 12, and 16 g L^−1^ and reported generally better growth and survival at 8 g L^−1^ than at the higher salinity levels [6]. However, *Nitraria tangutorum* Bobrov was not included in that assessment, and its physiological responses to comparable long-term saline irrigation remain insufficiently characterized.

Plants encounter multiple environmental constraints during growth and development, which can be broadly classified into biotic stresses, such as pathogen infection and herbivory, and abiotic stresses, such as drought, salinity, heat, cold, nutrient limitation, and heavy-metal toxicity [7]. Among these abiotic factors, salinity is particularly important in arid restoration systems because it can restrict water uptake, disturb ion homeostasis, inhibit photosynthesis, accelerate reactive oxygen species accumulation, and alter carbon and nitrogen metabolism, ultimately affecting plant growth, development, survival, yield formation, and plant quality [8]. Under field conditions, saline irrigation may therefore act not only as a water source but also as a chronic stress factor, especially in mining-disturbed substrates where poor soil structure, low fertility, and high evaporative demand can intensify salt accumulation around the root zone [9].

In addition to physiological and biochemical regulation, plant responses to salinity and other abiotic stresses are controlled by transcription factors and stress-responsive functional genes. Major transcription-factor families, including *NAC, WRKY*, and *MYB*, regulate downstream pathways associated with osmotic adjustment, antioxidant defense, abscisic acid signaling, ion homeostasis, and stress-related gene expression. For example, overexpression of *FvNAC29* from *Fragaria vesca* enhanced salt and cold tolerance in *Arabidopsis thaliana*; *MbWRKY50* from *Malus baccata* improved cold and drought tolerance by enhancing antioxidant capacity associated with reactive oxygen species scavenging; *MbWRKY2* improved drought tolerance in transgenic tobacco by regulating oxidative-stress and osmotic-adjustment-related responses; and *VhMYB15* from grape increased salinity and drought tolerance in *Arabidopsis thaliana* [10,11,12,13]. These studies indicate that physiological stress responses are often downstream manifestations of coordinated molecular regulation. Although the present study did not quantify transcription factors or gene expression, this molecular background provides an important context for interpreting the integrated physiological and biochemical responses of field-established *N. tangutorum* under long-term saline irrigation.

*Nitraria tangutorum* is a xerohalophytic shrub naturally distributed in western and northern China, including Xinjiang, and is well adapted to arid and saline environments [14,15,16]. Salinity can influence plant water relations, osmolyte accumulation, reactive oxygen species production, redox homeostasis, nitrogen metabolism, lipid turnover, and phytohormone signaling [17]. Reactive oxygen species are particularly important because they function in stress signaling at controlled levels but cause oxidative injury when their production exceeds cellular protective capacity [18]. The ascorbate–glutathione system represents a major component of cellular redox regulation, while nitrogen metabolism and phytohormonal coordination are also closely associated with plant adjustment to salinity [19]. Although these individual responses are well established, they are frequently investigated separately or under short-term controlled conditions.

For restoration-oriented studies, this creates an important knowledge gap. Field-established shrubs experience repeated exposure to mixed salts under naturally fluctuating soil moisture, temperature, radiation, and evaporative conditions, and their long-term physiological state may differ from that observed in short-term laboratory experiments. Moreover, increases in osmolytes, antioxidant-related proteins, or stress hormones do not necessarily indicate successful acclimation because such responses may occur simultaneously with oxidative injury, redox imbalance, and metabolic suppression. Integrated analysis of these pathways in both roots and shoots can therefore provide a more complete assessment of how *N. tangutorum* responds to increasing saline irrigation [20,21]. The significance of the present study lies in evaluating these coordinated responses under long-term field conditions rather than treating individual salt-stress responses as isolated indicators of tolerance.

Accordingly, this study investigated approximately two-year-old, field-established *N. tangutorum* plants maintained for approximately two growing seasons under freshwater, 8 g L^−1^, or 12 g L^−1^ irrigation-water total dissolved solids in the Dananhu mine-restoration area of Hami, Xinjiang. We examined pigments, osmolytes, oxidative-stress indicators, antioxidant-related proteins, AsA–GSH-cycle components, nitrogen-metabolism proteins, phytohormones, and triacylglycerols in roots and shoots. Specifically, the study aimed to: (i) quantify the magnitude and tissue-specific responses to increasing irrigation-water salinity; (ii) determine whether the 8 and 12 g L^−1^ treatments differed in the balance between stress-associated biochemical adjustment and physiological injury; and (iii) identify coordinated multi-pathway response patterns that can provide a physiological basis for subsequent studies incorporating ion homeostasis, growth, survival, and long-term field performance.

## 2. Results

### 2.1. Photosynthetic Pigments

Salinity induced contrasting changes in shoot total chlorophyll and carotenoid contents (Table 1). Total chlorophyll content decreased progressively from 3.92 ± 0.04 mg g^−1^ FW in the control to 3.36 ± 0.15 mg g^−1^ FW under the 8 g L^−1^ treatment and 3.04 ± 0.11 mg g^−1^ FW under the 12 g L^−1^ treatment. Relative to the control, these values represented reductions of 14.3% and 22.4%, respectively.

Shoot carotenoid content increased numerically from 54.46 ± 1.76 µg g^−1^ FW in the control to 58.87 ± 9.82 and 67.46 ± 6.46 µg g^−1^ FW under the 8 and 12 g L^−1^ treatments, respectively (Table 1). However, the differences among treatments were not statistically significant (*p* > 0.05). Thus, increasing irrigation-water salinity significantly reduced shoot total chlorophyll content, whereas carotenoid content showed no significant treatment effect.

### 2.2. Osmolytes and Total Free Amino Acids

**Figure 1:**
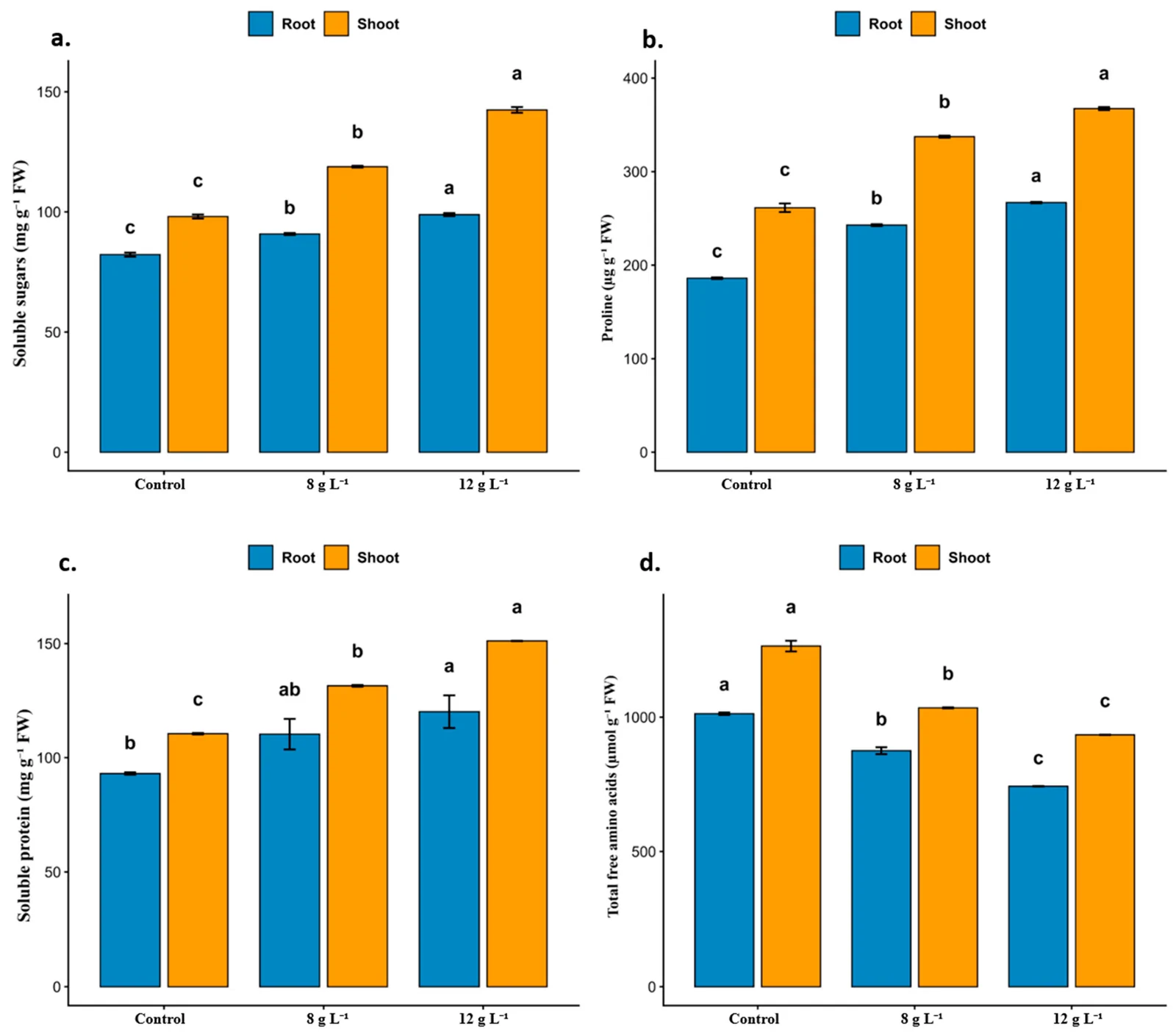
**Changes in osmolytes and total free amino acids in roots and shoots under salinity treatments.** (**a**) Soluble sugars; (**b**) proline; (**c**) soluble protein; and (**d**) total free amino acids. Bars represent the mean ± SE of three independent biological replicates (n = 3). Different lowercase letters indicate significant differences among salinity treatments within the same tissue according to Tukey’s honestly significant difference test at *p* < 0.05.

Salinity treatment significantly enhanced soluble sugar accumulation in both roots and shoots (Figure 1a). In roots, soluble sugar content increased from approximately 82 mg g^−1^ in the control to 91 mg g^−1^ at 8 g L^−1^ and 99 mg g^−1^ at 12 g L^−1^. In shoots, the increase was more pronounced, rising from approximately 98 mg g^−1^ to 119 mg g^−1^ and 142 mg g^−1^, respectively. Soluble sugar content increased significantly across the salinity gradient in both roots and shoots, with the highest values recorded under the 12 g L^−1^ treatment. Within each tissue, all three treatment means differed significantly from one another (*p* < 0.05).

Proline content increased significantly with increasing salinity in both roots and shoots (Figure 1b). In roots, proline content increased from approximately 185 µg g^−1^ in the control to 240 and 265 µg g^−1^ under the 8 and 12 g L^−1^ treatments, respectively. In shoots, proline content increased from approximately 260 µg g^−1^ in the control to 337 and 367 µg g^−1^ under the corresponding salinity treatments. Within each tissue, all three treatments differed significantly from one another according to Tukey’s honestly significant difference test (*p* < 0.05).

Protein content increased under salinity-induced stress, although the response differed slightly between roots and shoots (Figure 1c). In roots, protein content increased from approximately 93 mg g^−1^ in the control to 110 mg g^−1^ at 8 g L^−1^ and 120 mg g^−1^ at 12 g L^−1^. In roots, soluble protein content under the 8 g L^−1^ treatment did not differ significantly from either the control or the 12 g L^−1^ treatment, whereas the value under 12 g L^−1^ was significantly higher than that of the control (*p* < 0.05). In shoots, protein content increased more clearly from approximately 110 mg g^−1^ to 130 mg g^−1^ and 151 mg g^−1^, with distinct letters indicating significant differences among all treatments (*p* < 0.05).

Unlike soluble sugars, proline, and protein, total amino acid content declined with increasing irrigation-water salinity (Figure 1d). Total free amino-acid content decreased from approximately 1010 µmol g^−1^ FW in the control to 880 and 760 µmol g^−1^ FW in roots under the 8 and 12 g L^−1^ treatments, respectively, and from approximately 1300 µmol g^−1^ FW to 1030 and 930 µmol g^−1^ FW in shoots. Total free amino-acid content decreased significantly with increasing salinity in both roots and shoots (*p* < 0.05). Relative to the control, soluble sugars increased by 11.0% and 20.7% in roots and by 21.4% and 44.9% in shoots under the 8 and 12 g L^−1^ treatments, respectively. Proline increased by 29.7% and 43.2% in roots and by 29.6% and 41.2% in shoots, while soluble proteins increased by 18.3% and 29.0% in roots and by 18.2% and 37.3% in shoots. In contrast, total free amino acids decreased by 12.9% and 24.8% in roots and by 20.8% and 28.5% in shoots.

### 2.3. Oxidative Stress Indicators

**Figure 2:**
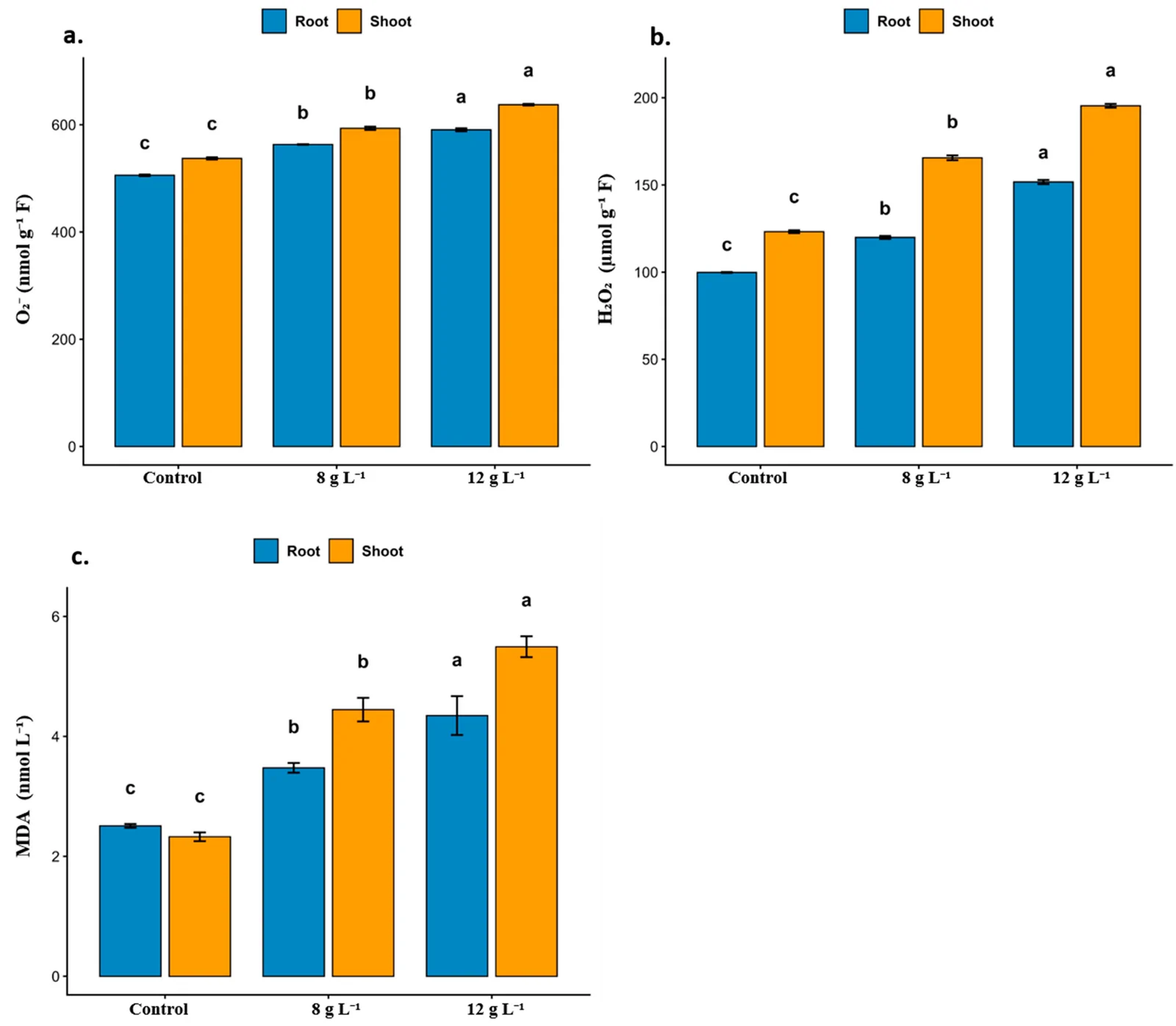
Effects of salinity treatments on oxidative stress indicators in roots and shoots. (**a**) Superoxide anion (O_2_^−^); (**b**) Hydrogen Peroxide (H_2_O_2_); (**c**) Malondialdehyde (MDA). Bars represent the mean ± SE of three independent biological replicates (n = 3). Different lowercase letters indicate significant differences among salinity treatments within the same tissue according to Tukey’s honestly significant difference test at *p* < 0.05.

Superoxide anion (O_2_^−^) levels increased significantly with increasing salinity in both roots and shoots (Figure 2a). In roots, O_2_^−^ levels increased from approximately 505 nmol g^−1^ FW in the control to 560 and 590 nmol g^−1^ FW under the 8 and 12 g L^−1^ treatments, respectively. In shoots, the corresponding values increased from approximately 535 nmol g^−1^ FW to 595 and 640 nmol g^−1^ FW. Within each tissue, all three treatment means differed significantly from one another according to Tukey’s honestly significant difference test (*p* < 0.05), indicating progressively greater O_2_^−^ accumulation with increasing salinity.

H_2_O_2_ content increased significantly with increasing salinity in both roots and shoots (Figure 2b). Relative to the control, H_2_O_2_ content increased by 21.0% and 51.0% in roots and by 34.7% and 57.3% in shoots under the 8 and 12 g L^−1^ treatments, respectively.

MDA content increased significantly with increasing salinity in both tissues, with a larger relative increase in shoots (Figure 2c). Root MDA increased from approximately 2.6 nmol L^−1^ in the control to 3.5 and 4.4 nmol L^−1^ under the 8 and 12 g L^−1^ treatments, respectively, whereas shoot MDA increased from approximately 2.4 to 4.5 and 5.4 nmol L^−1^. Relative to the control, O_2_^−^ increased by 10.9% and 16.8% in roots and by 11.2% and 19.6% in shoots under 8 and 12 g L^−1^, respectively. H_2_O_2_ increased by 21.0% and 51.0% in roots and by 34.7% and 57.3% in shoots, whereas MDA increased by 34.6% and 69.2% in roots and by 87.5% and 125.0% in shoots.

### 2.4. Antioxidant-Related Protein Concentrations

**Figure 3:**
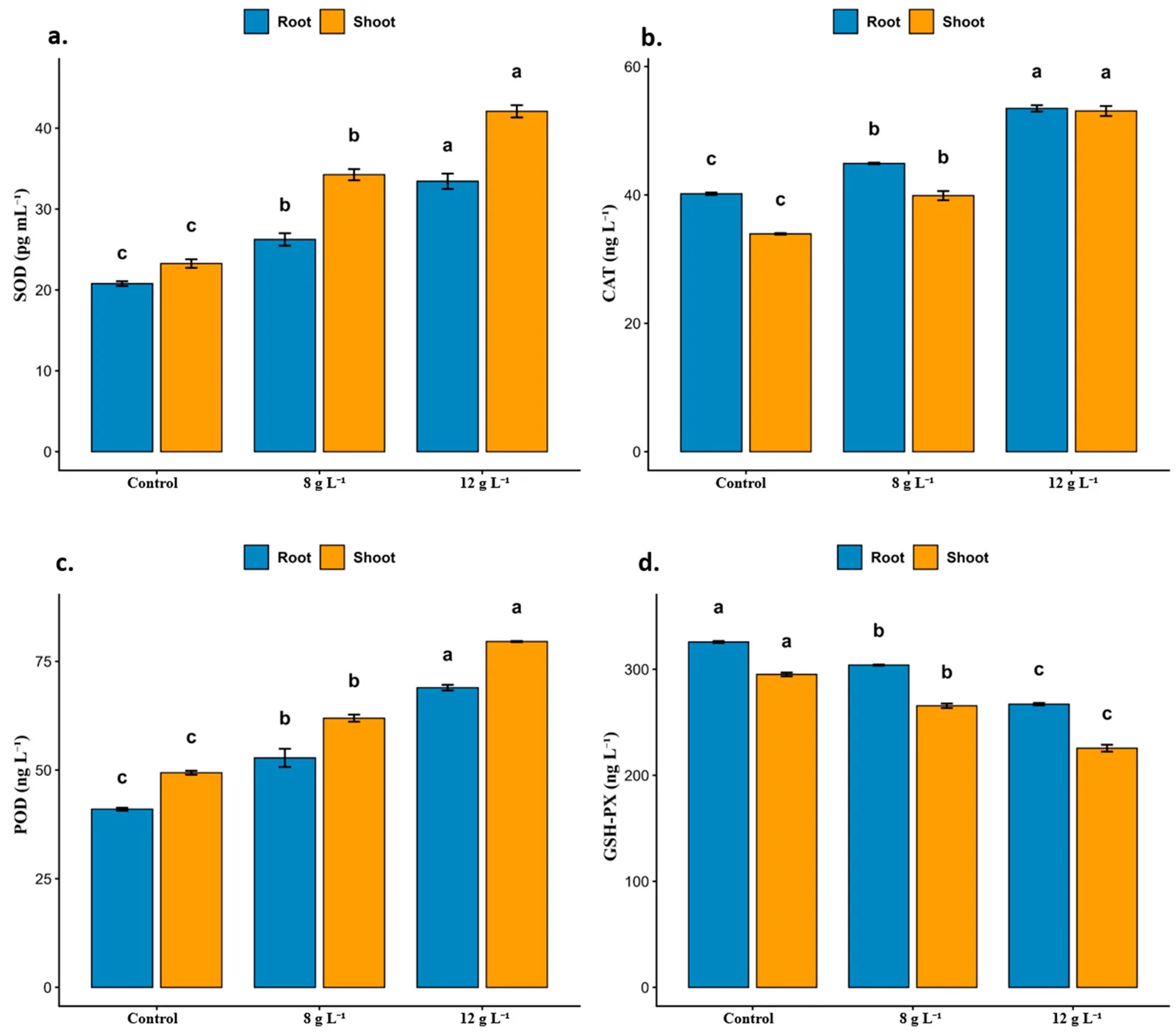
**Responses of antioxidant-related protein concentrations in roots and shoots under salinity treatments.** (**a**) Superoxide dismutase (SOD); (**b**) catalase (CAT); (**c**) peroxidase (POD); and (**d**) glutathione peroxidase (GSH-PX). Bars represent the mean ± SE of three independent biological replicates (n = 3). Different lowercase letters indicate significant differences among salinity treatments within the same tissue according to Tukey’s honestly significant difference test at *p* < 0.05.

Superoxide dismutase (SOD) protein concentration increased significantly with increasing salinity in both roots and shoots (Figure 3a). In roots, SOD concentration increased from approximately 21 pg mL^−1^ in the control to 27 and 34 pg mL^−1^ under the 8 and 12 g L^−1^ treatments, respectively. In shoots, the corresponding concentrations increased from approximately 24 pg mL^−1^ to 35 and 43 pg mL^−1^. The different lowercase letters indicate significant differences among the treatments within each tissue at *p* < 0.05. Because SOD was quantified using an immunochemical assay, these values represent SOD protein concentration rather than catalytic activity.

CAT protein concentration increased progressively with salinity in both roots and shoots (Figure 3b). Root CAT increased from approximately 40 ng L^−1^ in the control to 45 ng L^−1^ at 8 g L^−1^ and 53 ng L^−1^ at 12 g L^−1^. Shoot CAT increased from approximately 34 ng L^−1^ to 40 ng L^−1^ and 52 ng L^−1^, respectively. The different significance letters indicate that salt stress significantly enhanced CAT concentration (*p* < 0.05), especially under 12 g L^−1^.

POD protein concentration increased significantly with increasing salinity in both tissues (Figure 3c). In roots, POD increased from approximately 41 ng L^−1^ in the control to 53 ng L^−1^ at 8 g L^−1^ and 69 ng L^−1^ at 12 g L^−1^. In shoots, POD increased from approximately 49 ng L^−1^ to 62 ng L^−1^ and 79 ng L^−1^, respectively. The progressive letter changes indicate significant differences among all treatments (*p* < 0.05), demonstrating a progressive increase in POD protein concentration with increasing salinity.

Glutathione peroxidase (GSH-PX) detoxifies peroxides using glutathione-dependent reactions and is therefore linked to cellular redox regulation. In contrast to SOD, CAT, and POD, GSH-PX protein concentration decreased significantly with increasing salinity in both roots and shoots (Figure 3d). Root GSH-PX decreased from approximately 325 ng L^−1^ in the control to 305 ng L^−1^ at 8 g L^−1^ and 270 ng L^−1^ at 12 g L^−1^. Shoot GSH-PX decreased from approximately 295 ng L^−1^ to 265 ng L^−1^ and 228 ng L^−1^, respectively. The different significance letters indicate a significant decrease in GSH-PX protein concentration (*p* < 0.05), suggesting that not all antioxidant enzymes were equally maintained under increasing salinity.

Relative to the control, SOD protein concentration increased by 28.6% and 61.9% in roots and by 45.8% and 79.2% in shoots under the 8 and 12 g L^−1^ treatments, respectively. CAT increased by 12.5% and 32.5% in roots and by 17.6% and 52.9% in shoots, while POD increased by 29.3% and 68.3% in roots and by 26.5% and 61.2% in shoots. In contrast, GSH-PX decreased by 6.2% and 16.9% in roots and by 10.2% and 22.7% in shoots.

### 2.5. AsA-GSH Cycle

**Figure 4:**
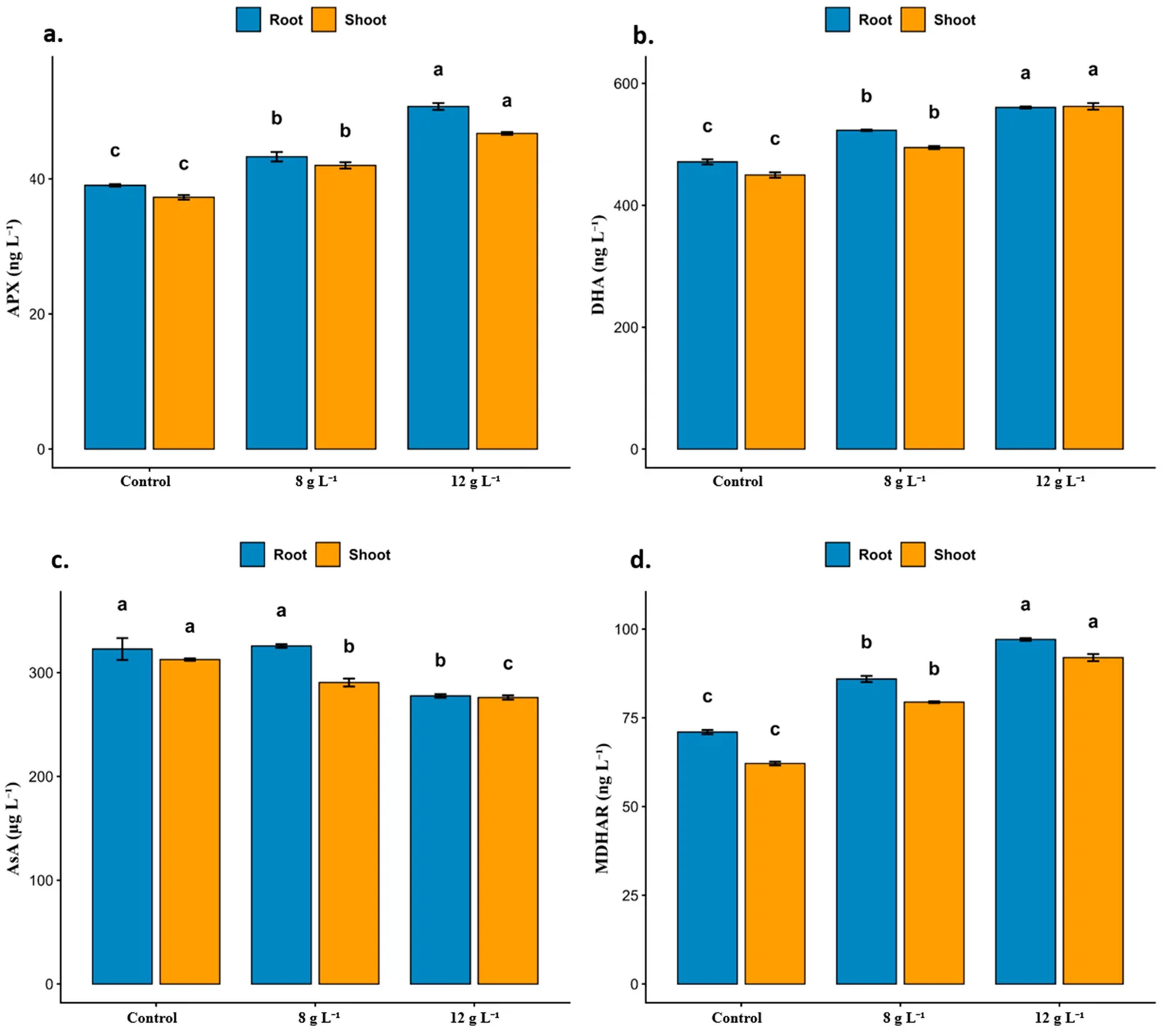

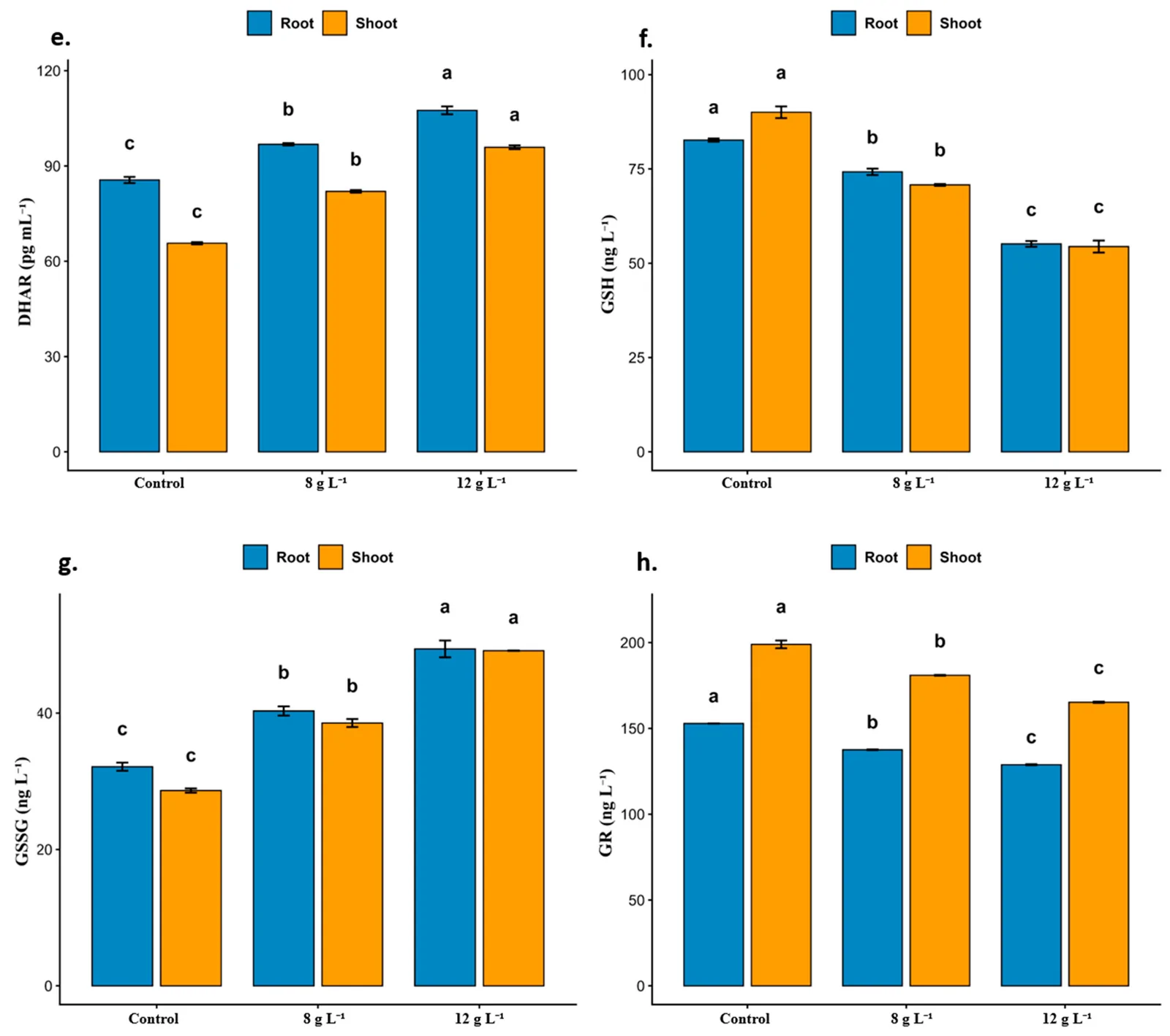
**Modulation of AsA–GSH cycle components in roots and shoots of *N. tangutorum* under salinity treatments.** (**a**) Ascorbate peroxidase (APX); (**b**) Dehydroascorbic acid (DHA; (**c**) Ascorbic acid/ascorbate (ASA); (**d**) Monodehydroascorbate reductase (MDHAR); (**e**) Dehydroascorbate reductase (DHAR); (**f**) Oxidized glutathione (GSH); (**g**) Reduced glutathione (GSSG); and (**h**) Glutathione reductase (GR). Bars represent the mean ± SE of three independent biological replicates (n = 3). Different lowercase letters indicate significant differences among salinity treatments within the same tissue according to Tukey’s honestly significant difference test at *p* < 0.05.

Ascorbate peroxidase (APX) concentration increased significantly with increasing irrigation-water salinity in both roots and shoots (Figure 4a). In roots, APX increased from approximately 39 ng L^−1^ in the control to 43 ng L^−1^ at 8 g L^−1^ and 52 ng L^−1^ at 12 g L^−1^. In shoots, APX increased from approximately 37 ng L^−1^ to 42 ng L^−1^ and 47 ng L^−1^, respectively. The distinct lowercase letters among treatments indicate significant differences within each tissue (*p* < 0.05), demonstrating a progressive increase in APX protein concentration under salinity.

Dehydroascorbic acid (DHA) content increased significantly with salt concentration in both tissues (Figure 4b). Root DHA increased from approximately 475 ng L^−1^ in the control to 525 ng L^−1^ at 8 g L^−1^ and 560 ng L^−1^ at 12 g L^−1^. Shoot DHA increased from approximately 455 ng L^−1^ to 495 ng L^−1^ and 560 ng L^−1^, respectively. The c–b–a significance pattern indicates that each salinity level differed significantly from the others (*p* < 0.05), revealing a progressive shift toward a more oxidized ascorbate status.

Ascorbic acid (AsA) content declined under salinity-induced stress, especially at the highest salinity level (Figure 4c). In roots, AsA remained relatively high in the control and 8 g L^−1^ treatments, at approximately 320–325 µg L^−1^, but decreased to approximately 275 µg L^−1^ at 12 g L^−1^. The shared letter between the control and 8 g L^−1^ treatment indicates no significant difference between these two treatments, whereas 12 g L^−1^ caused a significant reduction (*p* < 0.05). In shoots, AsA decreased from approximately 310 µg L^−1^ in the control to 290 µg L^−1^ at 8 g L^−1^ and 275 µg L^−1^ at 12 g L^−1^, with different letters indicating significant reductions across treatments (*p* < 0.05).

Monodehydroascorbate reductase (MDHAR) concentration increased markedly with increasing salinity (Figure 4d). In roots, MDHAR concentration increased from approximately 70 ng L^−1^ in the control to 86 ng L^−1^ at 8 g L^−1^ and 97 ng L^−1^ at 12 g L^−1^. In shoots, MDHAR concentration increased from approximately 62 ng L^−1^ in the control to 79 and 91 ng L^−1^ under the 8 and 12 g L^−1^ treatments, respectively. The distinct significance letters indicate significant increases among all treatments within both tissues (*p* < 0.05), confirming a progressive increase in MDHAR protein concentration with increasing salinity.

Dehydroascorbate reductase (DHAR) concentration increased progressively in response to salinity (Figure 4e). Root DHAR increased from approximately 86 pg mL^−1^ in the control to 96 pg mL^−1^ at 8 g L^−1^ and 107 pg mL^−1^ at 12 g L^−1^. Shoot DHAR increased from approximately 64 pg mL^−1^ to 82 pg mL^−1^ and 96 pg mL^−1^, respectively. The c–b–a significance pattern indicates significant enhancement of DHAR concentration at each salinity level (*p* < 0.05), demonstrating a progressive increase in DHAR protein concentration under salinity.

Oxidized glutathione (GSSG) content increased significantly with increasing irrigation-water salinity in both roots and shoots (Figure 4f). In roots, GSSG increased from approximately 32 ng L^−1^ in the control to 40 ng L^−1^ at 8 g L^−1^ and 50 ng L^−1^ at 12 g L^−1^. In shoots, GSSG increased from approximately 28 ng L^−1^ to 38 ng L^−1^ and 49 ng L^−1^, respectively. The distinct lowercase letters indicate significant differences among all treatments (*p* < 0.05), supporting a progressive oxidation of the glutathione pool under salinity.

Reduced glutathione (GSH) content declined significantly with increasing irrigation-water salinity (Figure 4g). In roots, GSH decreased from approximately 82 ng L^−1^ in the control to 74 ng L^−1^ at 8 g L^−1^ and 56 ng L^−1^ at 12 g L^−1^. In shoots, GSH decreased from approximately 88 ng L^−1^ to 70 ng L^−1^ and 55 ng L^−1^, respectively. The a–b–c significance pattern indicates significant depletion of the GSH pool under both the 8 and 12 g L^−1^ treatments (*p* < 0.05).

Glutathione reductase (GR) concentration decreased significantly with increasing salinity in both tissues (Figure 4h). In roots, GR decreased from approximately 153 ng L^−1^ in the control to 138 ng L^−1^ at 8 g L^−1^ and 129 ng L^−1^ at 12 g L^−1^. In shoots, GR decreased from approximately 198 ng L^−1^ to 181 ng L^−1^ and 165 ng L^−1^, respectively. The different significance letters indicate a significant decrease in GR protein concentration, showing a progressive decrease in GR protein concentration with increasing salinity.

Overall, salinity strongly altered the AsA–GSH system in *N. tangutorum*. The higher APX, MDHAR, and DHAR protein concentrations indicate increased abundance of several AsA–GSH-cycle-related proteins; however, catalytic recycling rates were not measured. Concurrently, AsA and GSH decreased while DHA and GSSG accumulated, indicating a shift toward a more oxidized cellular redox state. The decrease in GR protein concentration under the 12 g L^−1^ treatment occurred together with GSH depletion and GSSG accumulation, further supporting progressive oxidation of the glutathione pool. Taken together, the reciprocal decrease in the reduced antioxidant pools (AsA and GSH) and increase in their oxidized forms (DHA and GSSG) are consistent with increasingly oxidized redox conditions under salinity. AsA/DHA and GSH/GSSG ratios were not treated as independent experimental endpoints and are therefore not reported.

Relative to the control, APX increased by 10.3% and 33.3% in roots and by 13.5% and 27.0% in shoots at 8 and 12 g L^−1^, respectively. DHA increased by 10.5% and 17.9% in roots and by 8.8% and 23.1% in shoots; MDHAR increased by 22.9% and 38.6% in roots and by 27.4% and 46.8% in shoots; and DHAR increased by 11.6% and 24.4% in roots and by 28.1% and 50.0% in shoots. GSSG increased by 25.0% and 56.2% in roots and by 35.7% and 75.0% in shoots. Conversely, GSH decreased by 9.8% and 31.7% in roots and by 20.5% and 37.5% in shoots, while GR decreased by 9.8% and 15.7% in roots and by 8.6% and 16.7% in shoots. AsA changed little in roots at 8 g L^−1^ but decreased by approximately 14.1% at 12 g L^−1^; in shoots, it decreased by 6.5% and 11.3%, respectively.

### 2.6. Nitrogen Metabolism

**Figure 5:**
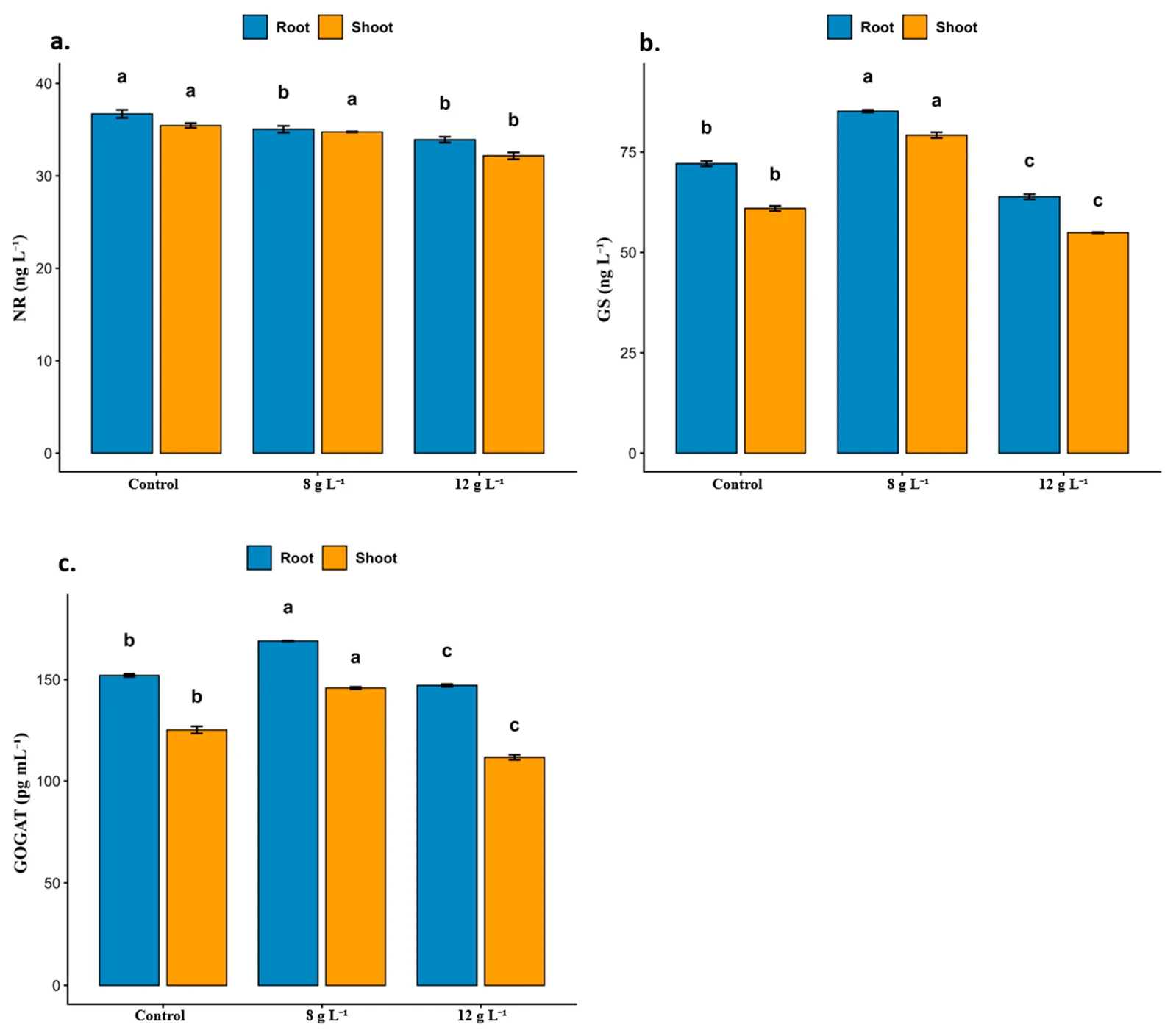
**Responses of nitrogen-metabolism-related protein concentrations in roots and shoots under salinity treatments.** (**a**) Nitrate reductase (NR); (**b**) glutamine synthetase (GS); and (**c**) glutamate synthase (GOGAT). Bars represent the mean ± SE of three independent biological replicates (n = 3). Different lowercase letters indicate significant differences among salinity treatments within the same tissue according to Tukey’s honestly significant difference test at *p* < 0.05.

Nitrate reductase (NR) levels decreased in response to salinity in both roots and shoots (Figure 5a). In roots, NR decreased from approximately 36.5 ng L^−1^ in the control to 35.0 and 34.0 ng L^−1^ under the 8 and 12 g L^−1^ treatments, respectively. NR levels under both salinity treatments were significantly lower than those in the control, whereas no significant difference was observed between the 8 and 12 g L^−1^ treatments. In shoots, NR levels were approximately 35.5, 34.7, and 32.0 ng L^−1^ under the control, 8 g L^−1^, and 12 g L^−1^ treatments, respectively. The control and 8 g L^−1^ treatments did not differ significantly, whereas both were significantly higher than the 12 g L^−1^ treatment (*p* < 0.05). These results indicate that the higher salinity treatment caused a more pronounced reduction in NR levels, particularly in shoots.

Glutamine synthetase (GS) concentration showed a biphasic response to salt treatment (Figure 5b). In roots, GS increased from approximately 72 ng L^−1^ in the control to 85 ng L^−1^ at 8 g L^−1^, but then declined to approximately 65 ng L^−1^ at 12 g L^−1^. In shoots, GS increased from approximately 62 ng L^−1^ to 79 ng L^−1^ at 8 g L^−1^, followed by a decrease to approximately 55 ng L^−1^ at 12 g L^−1^. The three treatments showed different letters, indicating a significant increase in GS protein concentration at 8 g L^−1^ followed by a significant decrease at 12 g L^−1^.

GOGAT concentration also displayed a biphasic response (Figure 5c). In roots, GOGAT increased from approximately 152 pg mL^−1^ in the control to 170 pg mL^−1^ at 8 g L^−1^, then decreased to approximately 148 pg mL^−1^ at 12 g L^−1^. In shoots, GOGAT increased from approximately 126 pg mL^−1^ to 146 pg mL^−1^ at 8 g L^−1^, followed by a marked decrease to approximately 112 pg mL^−1^ at 12 g L^−1^. The distinct letters indicate significant differences among treatments (*p* < 0.05), demonstrating a biphasic change in GOGAT protein concentration, with an increase at 8 g L^−1^ followed by a decrease at 12 g L^−1^. Relative to the control, NR decreased by 4.1% and 6.8% in roots and by 2.3% and 9.9% in shoots under the 8 and 12 g L^−1^ treatments, respectively. GS displayed a biphasic response, increasing by 18.1% in roots and 27.4% in shoots at 8 g L^−1^ but decreasing by 9.7% and 11.3%, respectively, at 12 g L^−1^. GOGAT similarly increased by 11.8% in roots and 15.9% in shoots at 8 g L^−1^ but was 2.6% and 11.1% below control values, respectively, at 12 g L^−1^.

### 2.7. Growth Hormones

**Figure 6:**
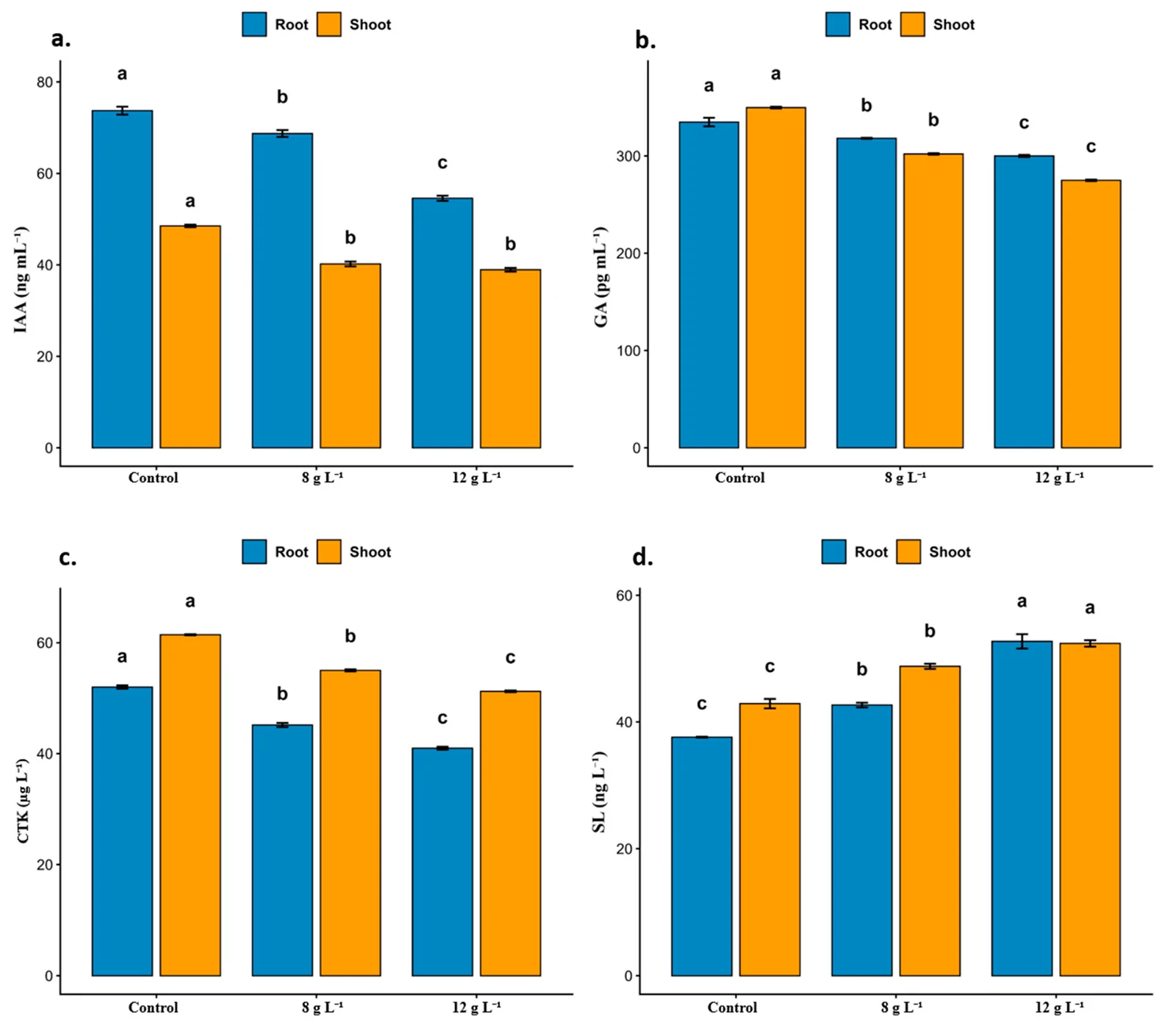
**Effects of salinity treatments on growth-related phytohormones in roots and shoots.** (**a**) Indole-3-acetic acid (IAA); (**b**) Gibberellic acid (GA); (**c**) Cytokinin (CTK); and (**d**) Strigolactone (SL). Bars represent the mean ± SE of three independent biological replicates (n = 3). Different lowercase letters indicate significant differences among salinity treatments within the same tissue according to Tukey’s honestly significant difference test at *p* < 0.05.

Indole-3-acetic acid (IAA) content decreased with increasing irrigation-water salinity (Figure 6a). In roots, IAA declined from approximately 74 µg L^−1^ in the control to 69 µg L^−1^ at 8 g L^−1^ and 55 µg L^−1^ at 12 g L^−1^, with different letters indicating significant differences among treatments (*p* < 0.05). In shoots, IAA decreased from approximately 49 µg L^−1^ in the control to 40 µg L^−1^ at 8 g L^−1^ and 39 µg L^−1^ at 12 g L^−1^. The salt treatments showed lower significance groups than the control, indicating significant inhibition of IAA accumulation under salinity.

Gibberellic (GA) content was significantly reduced by salinity (Figure 6b). In roots, GA decreased from approximately 330 pg mL^−1^ in the control to 315 pg mL^−1^ at 8 g L^−1^ and 300 pg mL^−1^ at 12 g L^−1^. In shoots, GA declined more strongly from approximately 347 pg mL^−1^ to 300 pg mL^−1^ and 278 pg mL^−1^, respectively. The different significance letters indicate that both salinity treatments significantly reduced GA levels (*p* < 0.05), with the strongest suppression under 12 g L^−1^.

Cytokinin (CTK) content decreased with increasing salinity in both roots and shoots (Figure 6c). Root CTK declined from approximately 51 µg L^−1^ in the control to 45 and 41 µg L^−1^ under the 8 and 12 g L^−1^ treatments, respectively. Shoot CTK decreased from approximately 61 µg L^−1^ in the control to 55 and 51 µg L^−1^, respectively. The treatments differed significantly within each tissue (*p* < 0.05).

Unlike IAA, CTK, and GA, strigolactone (SL) content increased under salinity-induced stress (Figure 6d). Root SL increased from approximately 38 ng L^−1^ in the control to 43 ng L^−1^ at 8 g L^−1^ and 52 ng L^−1^ at 12 g L^−1^. Shoot SL increased from approximately 43 ng L^−1^ to 48 ng L^−1^ and 51 ng L^−1^, respectively. The different letters indicate significant increases with increasing irrigation-water salinity (*p* < 0.05), suggesting that SL accumulation may be associated with adaptive hormonal reprogramming under salinity. Relative to the control, IAA decreased by 6.8% and 25.7% in roots and by 18.4% and 20.4% in shoots under 8 and 12 g L^−1^, respectively. CTK decreased by 11.8% and 19.6% in roots and by 9.8% and 16.4% in shoots, while GA declined by 4.5% and 9.1% in roots and by 13.5% and 19.9% in shoots. In contrast, SL increased by 13.2% and 36.8% in roots and by 11.6% and 18.6% in shoots.

### 2.8. Stress Hormones

**Figure 7:**
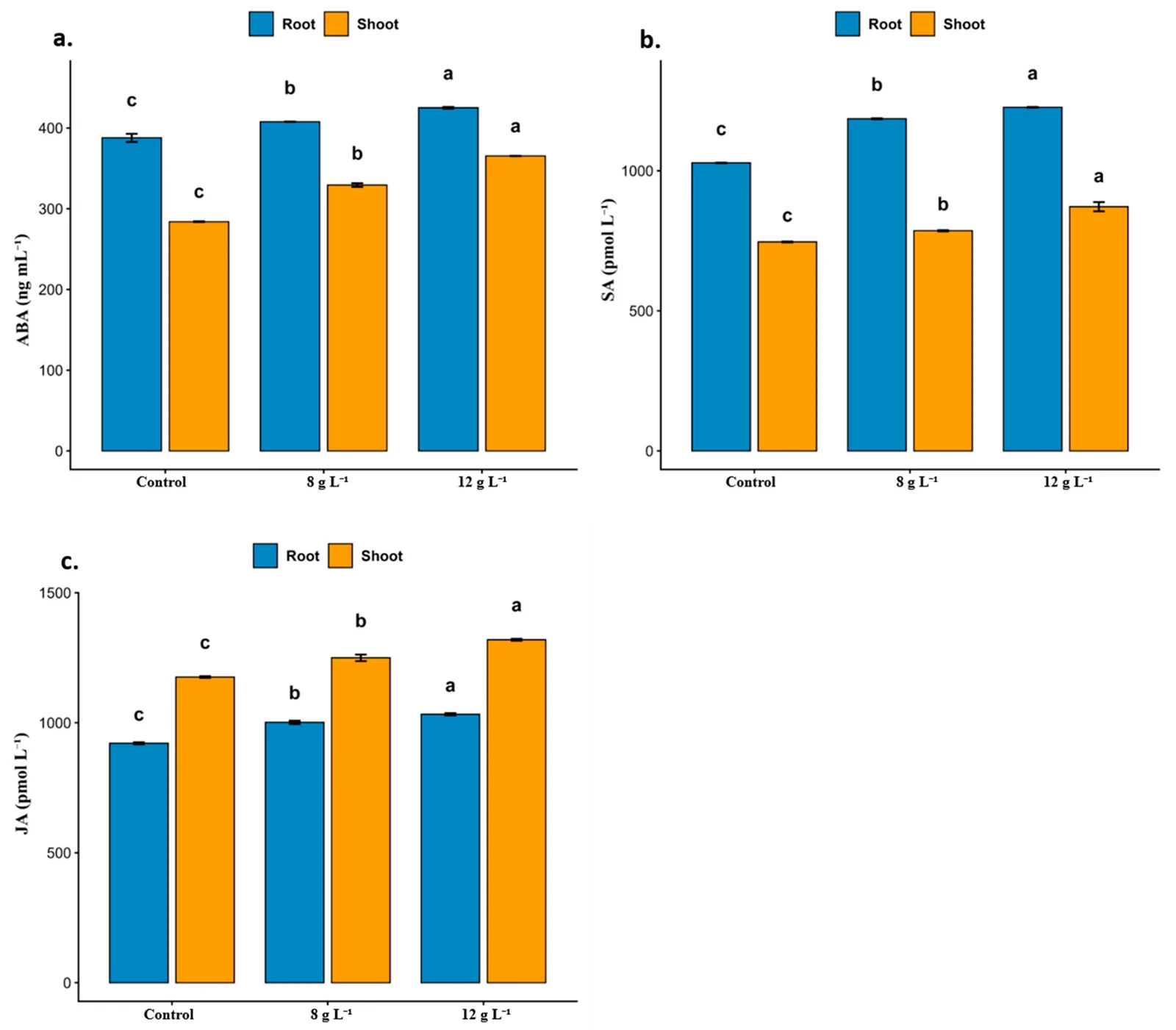
**Effects of salinity treatments on stress-related phytohormones in roots and shoots.** (**a**) Abscisic acid (ABA); (**b**) Salicylic acid (SA); and (**c**) Jasmonic acid (JA). Bars represent the mean ± SE of three independent biological replicates (n = 3). Different lowercase letters indicate significant differences among salinity treatments within the same tissue according to Tukey’s honestly significant difference test at *p* < 0.05.

Abscisic acid (ABA) content increased significantly with salt concentration in both roots and shoots (Figure 7a). In roots, ABA increased from approximately 390 µg L^−1^ in the control to 409 µg L^−1^ at 8 g L^−1^ and 425 µg L^−1^ at 12 g L^−1^. In shoots, ABA increased more strongly from approximately 285 µg L^−1^ to 330 µg L^−1^ and 365 µg L^−1^, respectively. The different significance letters indicate significant ABA accumulation under both salinity treatments (*p* < 0.05), with the highest level at 12 g L^−1^.

Salicylic acid (SA) content increased significantly with salt concentration (Figure 7b). In roots, SA increased from approximately 1025 pmol L^−1^ in the control to 1170 pmol L^−1^ at 8 g L^−1^ and 1245 pmol L^−1^ at 12 g L^−1^. In shoots, SA increased from approximately 745 pmol L^−1^ to 790 pmol L^−1^ and 860 pmol L^−1^, respectively. The different letters indicate significant salt-induced increases (*p* < 0.05), demonstrating activation of SA-associated defense signaling under salinity.

Jasmonic acid (JA) content increased progressively under salinity (Figure 7c). Root JA increased from approximately 920 pmol L^−1^ in the control to 1000 pmol L^−1^ at 8 g L^−1^ and 1040 pmol L^−1^ at 12 g L^−1^. Shoot JA increased from approximately 1160 pmol L^−1^ to 1230 pmol L^−1^ and 1310 pmol L^−1^, respectively. The distinct letters indicate significant differences among all treatments (*p* < 0.05), suggesting progressive activation of JA-mediated stress responses.

Relative to the control, ABA increased by 4.9% and 9.0% in roots and by 15.8% and 28.1% in shoots under the 8 and 12 g L^−1^ treatments, respectively. JA increased by 8.7% and 13.0% in roots and by 6.0% and 12.9% in shoots, while SA increased by 14.1% and 21.5% in roots and by 6.0% and 15.4% in shoots.

### 2.9. Lipid Metabolism

**Figure 8:**
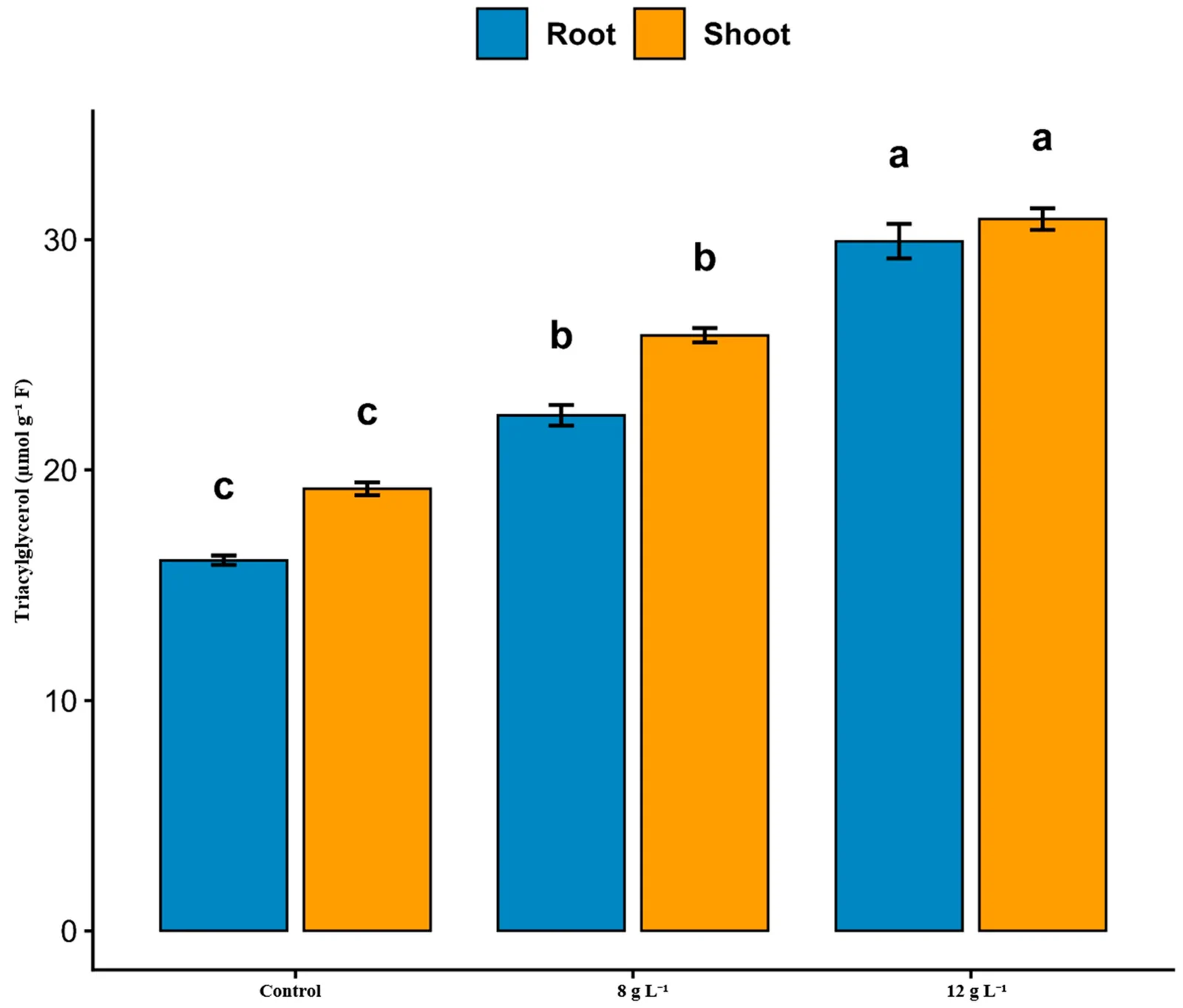
**Effects of salinity treatments on triacylglycerol (TAG) content in roots and shoots.** Bars represent the mean ± SE of three independent biological replicates (n = 3). Different lowercase letters indicate significant differences among salinity treatments within the same tissue according to Tukey’s honestly significant difference test at *p* < 0.05.

Triacylglycerol (TAG) content increased significantly with increasing salinity in both roots and shoots (Figure 8). In roots, TAG content increased from approximately 16 µmol g^−1^ FW in the control to 22.5 and 30 µmol g^−1^ FW under the 8 and 12 g L^−1^ treatments, respectively. In shoots, TAG content increased from approximately 19 µmol g^−1^ FW in the control to 26 and 31 µmol g^−1^ FW, respectively. Relative to the control, TAG content increased by 40.6% and 87.5% in roots and by 36.8% and 63.2% in shoots under the 8 and 12 g L^−1^ treatments, respectively.

### 2.10. Comparative PCA Analysis of Root and Shoot Traits

**Figure 9:**
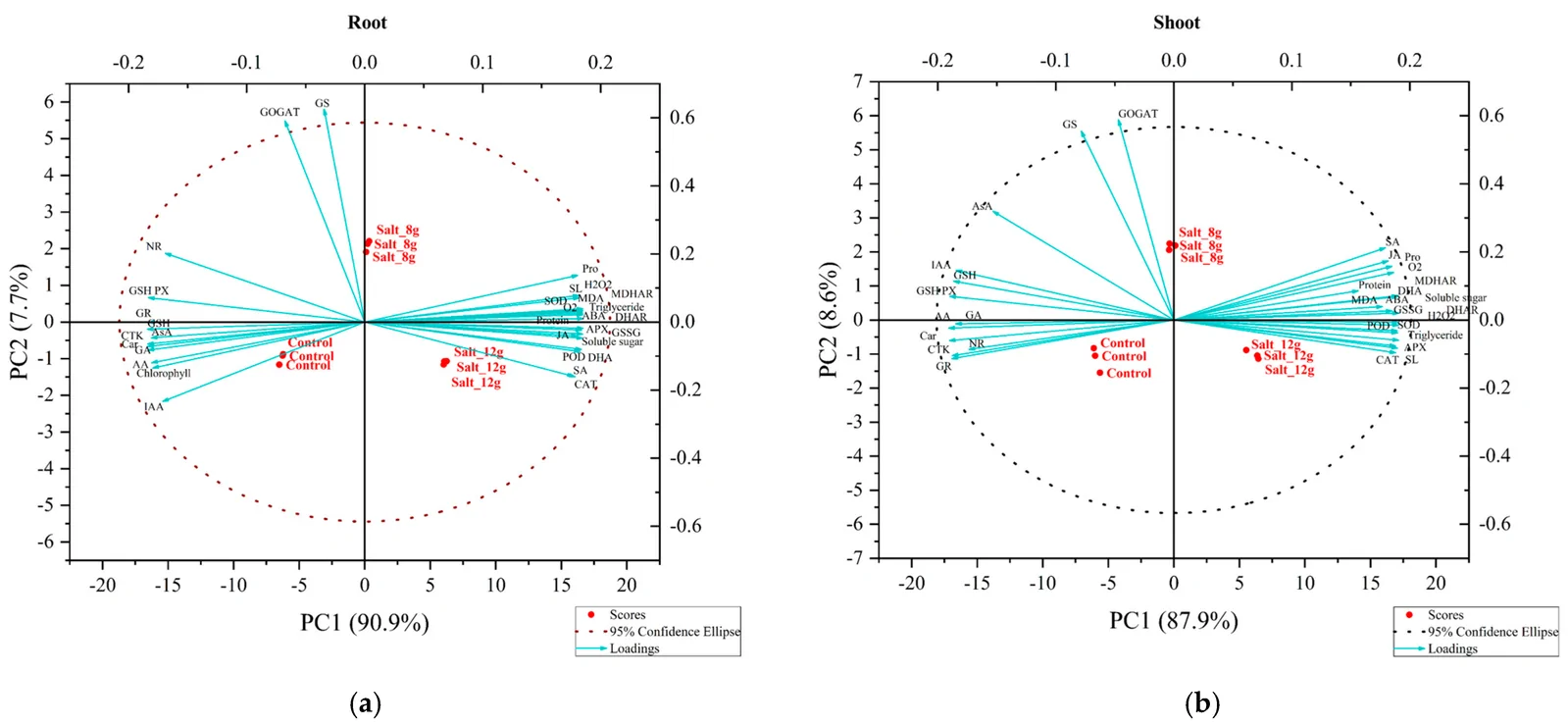
**Comparative principal component analysis (PCA) of physiological and biochemical traits in roots and shoots under salinity treatments.** PCA biplots show treatment scores and trait loadings for (**a**) roots and (**b**) shoots under the control, 8 g L^−1^, and 12 g L^−1^ treatments. PC1 and PC2 indicate the first and second principal components, respectively. Vectors indicate the direction and relative contribution of individual traits, and dotted circles represent the correlation circle.

Principal component analysis (PCA) revealed clear separation among the control, 8 g L^−1^, and 12 g L^−1^ treatments in both root and shoot tissues (Figure 9a,b). In roots, PC1 and PC2 explained 90.9% and 7.7% of the total variance, respectively, accounting for a cumulative variance of 98.6%. In shoots, PC1 and PC2 explained 87.9% and 8.6% of the total variance, respectively, accounting for a cumulative variance of 96.5%. In both tissue-specific PCAs, the control was positioned on the negative side of PC1 and was associated primarily with growth- and pigment-related traits, including IAA, GA, carotenoids, and NR, as well as chlorophyll in shoots. By contrast, the 12 g L^−1^ treatment was positioned on the positive side of PC1 and was associated with stress-responsive variables, including proline, soluble sugars, H_2_O_2_, MDA, ABA, JA, SA, and several antioxidant-related variables. The 8 g L^−1^ treatment was differentiated mainly along PC2 and was associated more closely with the nitrogen-assimilation variables GS and GOGAT. Overall, the PCA patterns indicate that increasing salinity shifted the physiological and biochemical profiles of both tissues from growth- and pigment-associated traits toward osmotic adjustment, stress-hormone accumulation, oxidative responses, and antioxidant-related regulation. Because PCA was conducted separately for roots and shoots, the difference in cumulative variance explained by the first two components should not be interpreted as evidence that salinity responses were stronger in one tissue than in the other.

### 2.11. Comparative Correlation Analysis of Root and Shoot Traits

**Figure 10:**
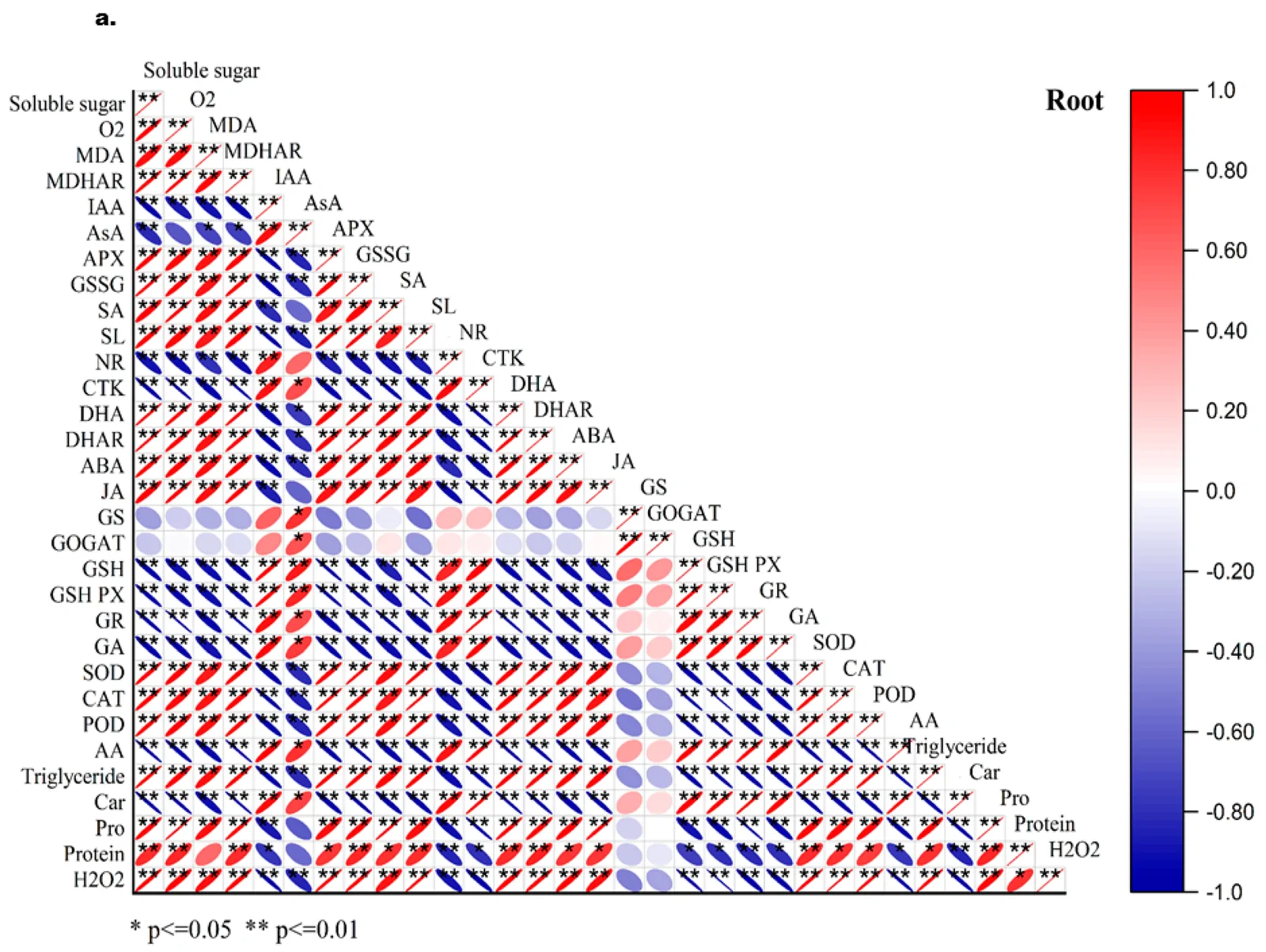

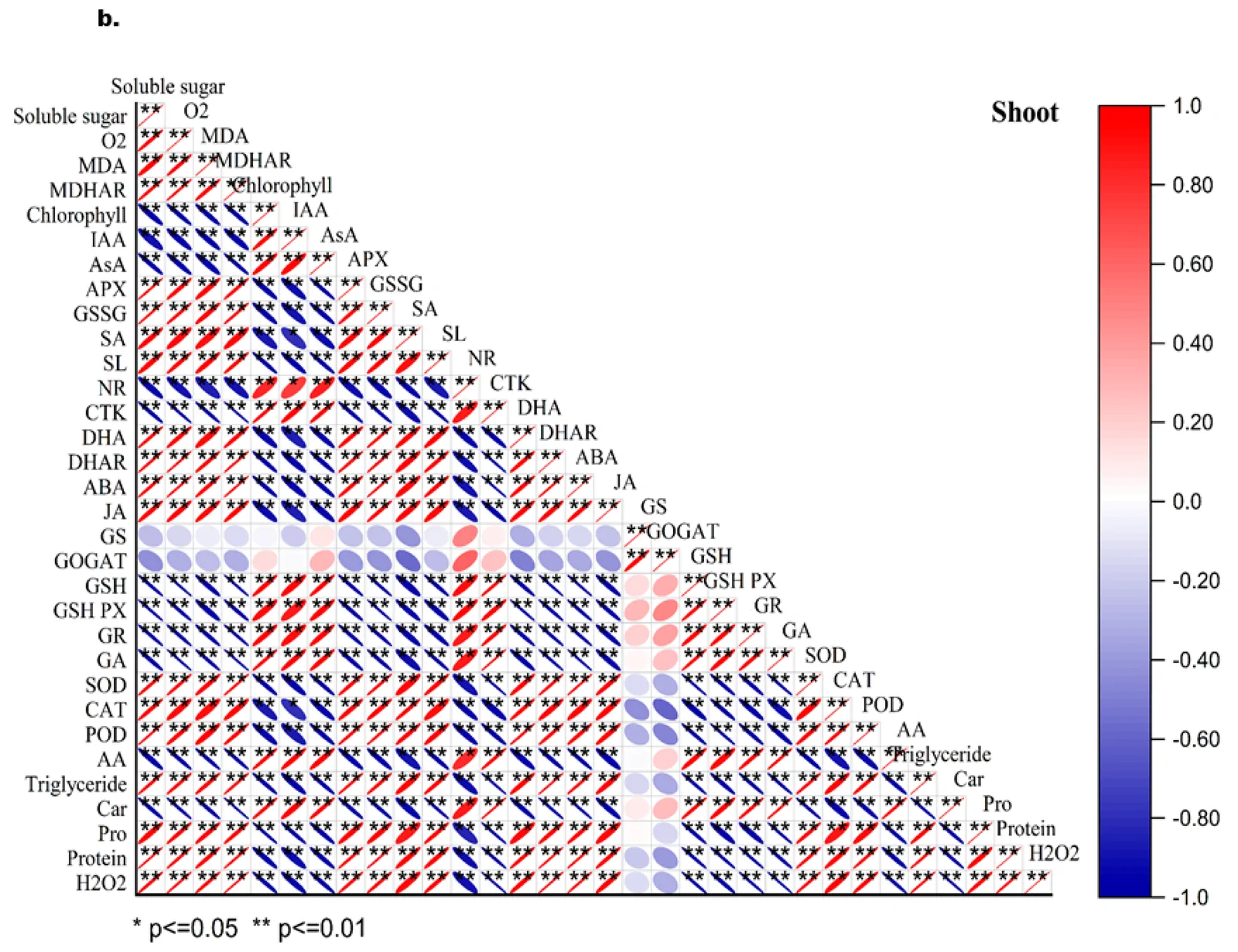
**Comparative correlation matrices of physiological and biochemical traits in roots and shoots under salinity treatments.** Pairwise Pearson correlation coefficients are shown for (**a**) roots and (**b**) shoots. Red and blue ellipses indicate positive and negative correlations, respectively, with darker colors and narrower ellipses representing stronger associations. Correlation coefficients range from −1 to 1. Statistical significance is based on false-discovery-rate-adjusted *p* values.

The correlation matrices revealed broadly similar relationships among the measured physiological and biochemical traits in roots and shoots (Figure 10a,b). In both tissues, soluble sugars, O_2_^−^, MDA, H_2_O_2_, proline, soluble protein, ABA, JA, SA, APX, SOD, CAT, and POD were predominantly positively correlated, indicating coordinated changes among osmolyte accumulation, stress-associated hormones, oxidative-stress indicators, and antioxidant-related protein concentrations under salinity. MDA and H_2_O_2_ also showed strong positive associations with several antioxidant-related proteins and stress-associated metabolites.

Conversely, IAA, GA, CTK, NR, AsA, GSH, GSH-PX, and GR generally showed negative correlations with oxidative-stress indicators and stress-associated variables. In shoots, total chlorophyll and carotenoids were also negatively associated with several oxidative-stress variables, with total chlorophyll showing particularly strong negative relationships with MDA and H_2_O_2_ and positive associations with IAA and AsA. GS and GOGAT showed weaker and less consistent relationships with several other measured traits; however, these correlations do not establish functional independence of nitrogen metabolism.

Overall, the correlation patterns indicate a shift from growth-associated hormones, pigment maintenance, and reduced antioxidant metabolites toward osmolyte accumulation, stress-associated hormonal signaling, oxidative injury, and altered antioxidant-related protein abundance under increasing salinity. Statistical significance was evaluated using false-discovery-rate-adjusted *p* values, with adjusted *p* < 0.05 considered significant.

## 3. Discussion

Plant responses to salinity involve a multilevel regulatory network that links osmotic adjustment, ion balance, reactive oxygen species production, antioxidant defense, redox homeostasis, nitrogen metabolism, phytohormone signaling, lipid remodeling, and transcriptional regulation. Therefore, the physiological traits measured in the present study should be interpreted as downstream indicators of a broader stress-regulatory system rather than as isolated markers of tolerance. The observed increases in osmolytes, reactive oxygen species, antioxidant-related proteins, oxidized AsA–GSH components, stress-related hormones, and triacylglycerols indicate that long-term saline irrigation reshaped multiple interconnected pathways in field-established *N. tangutorum*.

The individual responses observed here are broadly consistent with established plant responses to salinity, but the more informative feature of this dataset is their coordinated behavior under long-term field exposure. At 8 g L^−1^, osmolyte accumulation and increased GS/GOGAT protein abundance occurred together with measurable ROS and MDA accumulation, indicating that biochemical adjustment and oxidative injury coexisted under moderate saline irrigation. At 12 g L^−1^, stronger oxidative injury coincided with oxidation of the AsA and GSH pools, declining GSH-PX and GR protein concentrations, suppression of nitrogen-metabolism-related proteins, and stronger hormonal reprogramming. This pattern suggests a dose-dependent transition from partial physiological adjustment toward broader biochemical disruption.

Recent molecular studies further show that salinity and other abiotic stress responses are regulated by transcription factors and functional genes that coordinate antioxidant defense, osmotic adjustment, hormone signaling, and stress-related gene expression. For instance, *FvNAC29*, *MbWRKY50*, *MbWRKY2*, and *VhMYB15* have been shown to improve tolerance to salinity, drought, cold, or dehydration-related stresses by regulating downstream stress-responsive pathways [10,11,12,13]. Although transcription factors were not measured in the present study, these findings support the view that the biochemical traits observed in *N. tangutorum* represent downstream physiological outputs of broader molecular stress-regulatory networks. Thus, the present field study complements molecular stress studies by demonstrating how such downstream traits respond to chronic mixed-salt irrigation in a mining-restoration setting.

The individual responses observed in this study—osmolyte accumulation, ROS production, antioxidant-system modification, redox alteration, and hormonal changes—are broadly consistent with established plant responses to salinity and should not, by themselves, be considered novel. The more informative feature of the present dataset is its coordinated behavior under long-term field exposure. At 8 g L^−1^, increases in osmolytes and GS/GOGAT protein abundance occurred despite simultaneous ROS and MDA accumulation, whereas at 12 g L^−1^ stronger oxidative injury coincided with oxidation of the AsA and GSH pools, declining GSH-PX and GR, suppression of nitrogen-metabolism proteins, and stronger hormonal reprogramming. Thus, the field response was characterized by a dose-dependent transition from simultaneous adjustment and injury toward broader biochemical disruption [22].

This distinction is relevant to restoration-oriented salinity studies because most previous work has either evaluated field establishment and survival or examined individual physiological pathways under controlled salinity conditions. The present study bridges these approaches by characterizing multiple root and shoot pathways in established shrubs exposed repeatedly to mixed-salt irrigation under natural climatic and soil conditions.

### 3.1. Salinity Reduced Total Chlorophyll but Did Not Significantly Alter Shoot Carotenoids

Shoot total chlorophyll content declined progressively with increasing salinity, indicating that overall photosynthetic pigment maintenance became increasingly constrained under the tested conditions. The decline in total chlorophyll occurred concurrently with increasing ROS and MDA, supporting an association between pigment loss and oxidative injury. However, chlorophyll fluorescence, gas exchange, chloroplast ultrastructure, and pigment-degradation enzymes were not measured; therefore, the specific processes responsible for the observed reduction in total chlorophyll cannot be determined from the present data [23,24].

In contrast, shoot carotenoid content showed a numerical increase across the salinity treatments, but the differences were not statistically significant. The data therefore do not support carotenoid depletion or a significant salinity-induced change in carotenoid content [25,26]. The functional significance of the numerical increase cannot be established without measurements of carotenoid composition, photochemical efficiency, and non-photochemical quenching [27].

### 3.2. Osmolyte Accumulation Occurred Concurrently with Oxidative Injury

Soluble sugars, proline, and soluble proteins increased under salinity, whereas the total free amino acid pool declined. These changes demonstrate salinity-associated metabolic adjustment and selective accumulation of particular compatible solutes. Proline and soluble sugars can contribute to osmotic regulation, macromolecular stabilization, and redox protection; however, their accumulation alone does not demonstrate maintenance of cellular osmotic potential, tissue hydration, or turgor [28].

Importantly, osmolyte accumulation occurred concurrently with increases in O_2_^−^, H_2_O_2_, and MDA and with a reduction in shoot total chlorophyll. Therefore, the accumulation of these metabolites did not prevent oxidative injury under the tested salinity conditions. Because tissue water potential, osmotic potential, relative water content, turgor, growth, and survival were not measured, these responses are more appropriately interpreted as stress-associated osmolyte accumulation rather than evidence of successful osmotic compensation [29].

The simultaneous increase in proline and the decline in total free amino acids may reflect a selective redistribution of the amino acid pool toward particular stress-associated metabolites. However, individual amino acid profiles and metabolic fluxes were not measured; therefore, this interpretation remains a hypothesis requiring further validation [30].

### 3.3. ROS Accumulation and MDA Elevation Confirm Oxidative Injury Under High Salinity

The significant increases in O_2_^−^, H_2_O_2_, and MDA demonstrate that salinity generated oxidative stress in both roots and shoots. Under salinity-induced stress, ROS are commonly produced through impaired photosynthetic electron transport, mitochondrial respiration, plasma membrane NADPH oxidases, and peroxisomal metabolism. At low or moderate levels, ROS can function as signaling molecules, but excessive ROS damages proteins, lipids, carbohydrates, DNA, and cellular membranes [31].

The increase in MDA is particularly diagnostic because MDA is a final product of lipid peroxidation and reflects oxidative deterioration of membrane lipids. In the present study, MDA increased strongly at 12 g L^−1^, indicating that antioxidant protection was not sufficient to fully prevent membrane damage. This finding is physiologically consistent with the observed pigment loss: chloroplast membranes are highly sensitive to ROS, and oxidative injury to thylakoid membranes can accelerate chlorophyll degradation and reduce photosynthetic efficiency. Thus, the combined increase in ROS and MDA provides strong evidence that high salinity caused oxidative damage rather than only osmotic adjustment [32].

### 3.4. Antioxidant-Enzyme Protein Abundance Increased, but Oxidative Injury Persisted

The ELISA measurements showed that the immunoreactive protein concentrations of SOD, CAT, POD, and APX increased under salinity. Because ELISA quantifies antigen abundance rather than catalytic reaction rate, these increases should not be interpreted as direct evidence that enzyme activities or ROS-scavenging fluxes increased. They instead indicate that salinity was associated with increased abundance of several antioxidant-related proteins [33,34].

The concurrent accumulation of H_2_O_2_ and MDA demonstrates that these changes were insufficient to prevent oxidative injury, particularly under the 12 g L^−1^ treatment. Moreover, GSH-PX and GR protein concentrations declined as salinity increased, indicating that different antioxidant-system components responded differently. Collectively, the data show stress-associated restructuring of antioxidant protein abundance rather than uniformly enhanced antioxidant activity [35].

### 3.5. AsA–GSH-Pool Oxidation Exceeded the Capacity to Maintain the Reduced State

Salinity increased the protein concentrations of APX, MDHAR, and DHAR, while AsA and GSH declined and DHA and GSSG accumulated. This opposing response indicates that increased abundance of several AsA–GSH-cycle proteins were not sufficient to maintain the antioxidant pools in a reduced state. The decline in GR concentration is particularly relevant because GR supports the regeneration of GSH from GSSG; lower GR abundance may therefore have contributed to GSH depletion and GSSG accumulation [36,37].

The combined changes are consistent with a progressive shift of the ascorbate and glutathione pools toward a more oxidized state as salinity increased. However, ELISA-derived protein concentrations do not provide catalytic activities or metabolic fluxes. Accordingly, the results support progressive redox imbalance but do not demonstrate increased enzymatic recycling rates [31]. AsA/DHA and GSH/GSSG ratios calculated from individual biological replicates would provide a more direct quantitative description of redox-state change [38].

### 3.6. Biphasic Changes in GS and GOGAT Protein Abundance

NR protein concentration declined under salinity, whereas GS and GOGAT concentrations increased at 8 g L^−1^ and subsequently decreased at 12 g L^−1^. The higher GS and GOGAT concentrations at 8 g L^−1^ may reflect increased demand for ammonium assimilation or reassimilation and for glutamate supply to stress-related metabolic pathways. However, the simultaneous decline in the total free amino acid pool indicates that increased protein abundance did not necessarily correspond to greater net nitrogen assimilation [33].

At 12 g L^−1^, the concurrent reductions in NR, GS, GOGAT, and total free amino acids were consistent with broader disruption of nitrogen metabolism. These changes occurred together with stronger ROS accumulation, redox-pool oxidation, and chlorophyll loss, suggesting that increasing oxidative and metabolic pressure constrained the maintenance of nitrogen-related proteins. Because nitrate uptake, ammonium concentration, individual amino acids, enzyme activities, gene expression, and metabolic fluxes were not measured, the observed biphasic pattern should not be described as demonstrating compensatory nitrogen assimilation [39].

Previous studies of nitrogen metabolism under salinity indicate that nitrogen assimilation may be maintained or transiently stimulated under lower stress but becomes constrained as stress intensity increases [35,36]. The present biphasic GS/GOGAT pattern is consistent with this intensity-dependent framework, but differs from a simple monotonic stress response because the increase at 8 g L^−1^ occurred while NR and the total free amino acid pool were already declining. This suggests that the transient increase in GS/GOGAT protein abundance at 8 g L^−1^ reflected an intensity-dependent nitrogen-metabolic adjustment rather than improved whole-plant nitrogen status.

### 3.7. Hormonal Changes Were Consistent with, but Did Not Demonstrate, a Growth–Defense Shift

IAA, CTK, and GA declined with increasing salinity. These hormones are closely associated with cell elongation, cell division, root and shoot development, and developmental progression. Their coordinated decline is therefore consistent with reduced growth-associated hormonal signaling under salinity. However, biomass production, elongation rates, carbon allocation, and other direct measures of growth investment were not determined, and the hormonal changes alone cannot demonstrate a reallocation of resources from growth to defense. The observed pattern is consistent with previous studies showing that increasing salinity is frequently associated with decreases in auxins, cytokinins, and gibberellins [40,41].

In contrast, ABA, JA, SA, and SL increased under salinity. ABA is a central regulator of osmotic-stress signaling and stomatal responses, while JA and SA participate in stress-responsive signaling networks and interact with other phytohormonal pathways. Increased SL may also be associated with salinity-related changes in root development and resource acquisition. Thus, the combined hormonal profile is consistent with enhanced stress-associated signaling under increasing salinity, but does not by itself demonstrate a growth–defense trade-off [42]. The concurrent increase in stress-associated hormones together with total chlorophyll loss and MDA accumulation further indicates that the plants experienced substantial physiological stress, particularly under the 12 g L^−1^ treatment.

### 3.8. Triacylglycerol Accumulation Indicates Altered Lipid Metabolism Under Salinity

TAG content increased progressively with irrigation-water salinity in both roots and shoots, indicating substantial alteration of lipid metabolism under saline irrigation. TAG accumulation in vegetative tissues has been associated with lipid remodeling and the sequestration of excess fatty acids under abiotic stress [43]. In the present study, increasing TAG content occurred concurrently with increased MDA, indicating that changes in neutral-lipid storage coincided with greater lipid peroxidation. However, these measurements do not establish a direct mechanistic relationship between TAG accumulation and membrane damage. In particular, the present data cannot determine whether the accumulated TAG originated from membrane-lipid turnover, de novo fatty-acid synthesis, or other lipid-remodeling processes. TAG accumulation should therefore be interpreted as evidence of altered lipid metabolism under increasing salinity rather than direct evidence of either adaptive lipid remodeling or membrane-derived lipid sequestration. Targeted lipidomic analyses are required to distinguish among these possible sources and pathways [44].

### 3.9. Integrated Physiological Response and Study Limitations

At 8 g L^−1^, osmolyte accumulation and higher GS and GOGAT protein concentrations occurred concurrently with increased ROS and MDA. This combination indicates that stress-associated biochemical adjustment and oxidative injury occurred simultaneously rather than demonstrating successful physiological compensation. At 12 g L^−1^, greater chlorophyll loss, ROS and MDA accumulation, depletion of reduced antioxidant pools, lower GR and GSH-PX concentrations, and suppression of nitrogen-metabolism proteins indicated a broader loss of biochemical homeostasis [45].

Across the measured pathways, increasing antioxidant-related protein abundance did not prevent oxidation of the AsA and GSH pools. At the same time, the biphasic GS/GOGAT response and the opposing changes in growth- and stress-associated hormones indicate that redox regulation, nitrogen metabolism, and hormonal signaling responded jointly to increasing salinity. These coordinated changes are consistent with increasing metabolic burden under the higher salinity treatment [46].

Comparison with previous salinity literature further clarifies the contribution of the present study [47,48]. Controlled studies of halophytes, including species of *Nitraria*, have previously documented physiological and biochemical adjustment under increasing salinity, while broader studies have established the roles of osmolytes, ROS regulation, the AsA-GSH cycle, nitrogen metabolism, and phytohormone signaling in salt responses. Our findings therefore confirm rather than redefine these individual mechanisms. The added value of the present field experiment is that these pathways were evaluated simultaneously after long-term repeated exposure to mixed-salt irrigation, revealing that apparently protective responses did not necessarily indicate successful acclimation: osmolyte and antioxidant-related responses coexisted with oxidative injury at 8 g L^−1^, and the 12 g L^−1^ treatment was associated with coordinated deterioration across redox and nitrogen-metabolic components. This integrated pattern provides a more cautious framework for interpreting biochemical “stress responses” in restoration plants, where induction of protective-associated traits should not automatically be equated with physiological tolerance [49].

However, the present study did not determine tissue Na^+^, Cl^−^, K^+^, or Ca^2+^ concentrations, K^+^/Na^+^ or Ca^2+^/Na^+^ ratios, plant water status, biomass, survival, gas exchange, chlorophyll fluorescence, or recovery after stress removal. Consequently, the relative contributions of osmotic stress, Na^+^ or Cl^−^ toxicity, and nutrient imbalance cannot be distinguished. The measured variables characterize downstream physiological and biochemical responses but do not independently establish field tolerance, an irrigation threshold, or ecological-restoration suitability.

## 4. Materials and Methods

### 4.1. Study Area

The field experiment was conducted in an artificially restored coal-mining area in Dananhu Township, Yiwu District, Hami City, Xinjiang Uygur Autonomous Region, northwestern China (42°19′ N, 93°09′ E). The region has a temperate continental arid climate, with a mean annual temperature of approximately 10 °C, annual precipitation of 50.78 mm, annual evaporation of 3064.13 mm, and approximately 3358 h of sunshine per year. Mean annual relative humidity ranges from 39.5% to 50.7%, and the frost-free period is approximately 182 days.

The field substrate is characterized by saline-alkaline conditions and low nutrient availability. Before establishment of the restoration experiment, the soil contained 1.17 ± 0.08 g kg^−1^ organic matter, 0.09 ± 0.01 g kg^−1^ total nitrogen, 0.29 ± 0.03 g kg^−1^ total phosphorus, 34.1 ± 2.5 g kg^−1^ total potassium, 0.0313 ± 0.0025 g kg^−1^ hydrolysable nitrogen, 0.00217 ± 0.0003 g kg^−1^ available phosphorus, and 0.0613 ± 0.0042 g kg^−1^ available potassium. Low precipitation, strong evaporation, high solar radiation, soil salinity, and limited nutrient availability jointly constrain natural vegetation recovery in this region.

### 4.2. Plant Material, Salinity Treatments, and Tissue Sampling

#### 4.2.1. Plant Material and Field Growth Conditions

The broader restoration platform contained several desert shrub species established under the same field-management system. The present study specifically investigated *N. tangutorum*. The experimental plants were pre-established individuals from the long-term ecological-restoration plots and were designated as *N. tangutorum* in the original field experimental records. Because these plants were established as part of the restoration experiment rather than collected as wild botanical specimens, a separate voucher specimen was not prepared at the time of establishment or sampling, and no herbarium accession number is available. Plants were established directly in the field at a spacing of 1 m × 1 m, corresponding to a theoretical planting density of approximately 1.0 × 10^4^ plants ha^−1^, and were maintained under natural climatic and soil conditions using drip irrigation.

Root and shoot tissues were collected in June 2025 during the active-growth period. At the time of sampling, the field-established *N. tangutorum* plants were approximately two years old. Plants exhibiting mechanical injury, visible disease symptoms, severe herbivory, or atypical growth were excluded from sampling.

#### 4.2.2. Salinity-Treatment and Drip-Irrigation Design

A single-factor field experiment was conducted with irrigation water salinity as the experimental factor. The *N. tangutorum* restoration area comprised nine hydraulically independent plots, with three plots assigned to each of three irrigation treatments: a freshwater control, 8 g L^−1^ total dissolved solids, and 12 g L^−1^ total dissolved solids. Each independently irrigated plot constituted one experimental unit and one biological replicate.

The freshwater treatment contained no added saline mine water and served as the control. The saline irrigation waters were prepared by diluting highly mineralized mine water with freshwater until target total dissolved solids concentrations of 8 and 12 g L^−1^ were obtained. The source mine water was a mixed-salt water dominated by Na^+^, Cl^−^, SO_4_^2−^, and Ca^2+^ rather than a single NaCl solution. Total dissolved solids and electrical conductivity were measured before irrigation to verify the target concentrations. Separate storage reservoirs and irrigation pipelines were used for each treatment to prevent mixing among salinity levels.

The target salinity levels were applied directly through the drip-irrigation system and were not established through a stepwise increase. Drip irrigation was conducted throughout the growing season at an average interval of approximately seven days, with an irrigation amount of 148.15 m^3^ ha^−1^ per irrigation event. All plots received the same irrigation method, frequency, duration, and water volume; irrigation-water salinity was the only experimentally varied factor. Plants were maintained under their assigned salinity regimes from establishment until sampling in June 2025. Thus, the treatments represented long-term field exposure extending over approximately two growing seasons rather than a short-term salt-shock treatment.

#### 4.2.3. Tissue Sampling

Three healthy plants of comparable size and developmental stage were selected from the interior of each independently irrigated plot. Equal amounts of tissue from the selected plants were pooled to form one plot-level composite biological replicate. Consequently, each salinity treatment comprised three independent biological replicates corresponding to the three independently irrigated plots.

Fully developed current-season shoots were collected from comparable canopy positions. Fine roots with diameters below approximately 2 mm were excavated from the wetted root zone at a soil depth of approximately 20 cm. Root samples were rinsed briefly with clean water followed by deionized water to remove adhering soil and surface salts. Shoot samples were cleaned to remove dust and visible salt deposits. Excess surface moisture was removed with absorbent paper. Root and shoot samples were separated, immediately frozen in liquid nitrogen, transported on dry ice, and stored at −80 °C until analysis.

### 4.3. Sample Preparation

Frozen root and shoot tissues were ground separately to a fine powder in liquid nitrogen. Independent tissue aliquots were used for the different physiological and biochemical determinations to avoid repeated freezing and thawing.

For ELISA-based measurements, 0.50 g of frozen tissue powder was homogenized in 5.0 mL of ice-cold phosphate-buffered saline at pH 7.4. Sodium azide was not included because it inhibits the horseradish POD conjugate used in the immunoassays. Homogenates were centrifuged at 12,000× *g* for 15 min at 4 °C, and the clear supernatants were collected and analyzed promptly. Extracts were further diluted with the kit-specific sample diluent when their absorbance values exceeded the upper limit of the corresponding standard curve.

The commercial plant-specific double-antibody sandwich ELISA kits used to quantify MDA, antioxidant-related proteins, AsA–GSH-cycle components, and nitrogen-metabolism-related proteins were supplied by Shanghai Enzyme-linked Biotechnology Co., Ltd. (mlBio, Shanghai, China). The archived kit documentation retained the analyte-specific analytical ranges, reporting units, sample-dilution procedure, and assay conditions but did not include the catalog or lot numbers. The available product information is summarized in Table S1. All ELISA-based determinations were performed according to the corresponding manufacturer’s protocols and standard ELISA principles [50].

### 4.4. Determination of Photosynthetic Pigments

#### 4.4.1. Total Chlorophyll

Total chlorophyll content was determined spectrophotometrically following extraction with 80% acetone according to the method of Lichtenthaler and Wellburn, with minor modifications [51]. Approximately 0.20 g of fresh shoot tissue was homogenized in 10 mL of chilled 80% acetone under reduced light and centrifuged at 10,000× *g* for 10 min at 4 °C. Absorbance of the clear supernatant was measured at 663.2 and 646.8 nm using 80% acetone as the blank. Total chlorophyll concentration in the extract was calculated as:

$$C_{Chl,total}=7.15A_{663.2}+18.71A_{646.8}$$

where *A*_663.2_ and *A*_646.8_ are the absorbance values measured at 663.2 and 646.8 nm, respectively, and Chl, total is the total chlorophyll concentration in the extract (µg mL^−1^). Total chlorophyll content was calculated as Chl, total ×V/(1000W), where V is the extraction volume (mL) and W is the fresh tissue mass (g), and was expressed as mg g^−1^ FW. Chlorophyll a and chlorophyll b were not treated as independent physiological response variables in the present study.

#### 4.4.2. Total Carotenoids

Total carotenoids in shoot tissues were determined from the same 80% acetone extracts used for chlorophyll measurement according to the method of Lichtenthaler and Wellburn, with minor modifications [51]. Absorbance was measured at 470, 663.2, and 646.8 nm. Chlorophyll a and chlorophyll b concentrations were first calculated as intermediate computational terms:

$$C_{a}=12.25A_{663.2}-2.97A_{646.8}$$

$$C_{c}=21.50A_{646.8}-5.10A_{663.2}$$

Total carotenoid concentration was then calculated after correction for chlorophyll interference as:

$$C_{x+c}=\frac{100A_{470.2}-1.82C_{a}-85.02C_{b}}{198}$$

where *C_a_*, *C_b_*, and *C_x+c_* is expressed as µg mL^−1^ and *A*_470_, *A*_663.2_, and *A*_646.8_ represent absorbance at the corresponding wavelengths. Carotenoid content was calculated as *C_x+c_* × V/W, where V is the extraction volume (mL), and W is the fresh tissue mass (g), and was expressed as µg g^−1^ FW. Chlorophyll a and chlorophyll b were used solely as intermediate computational terms and were not analyzed as independent physiological response variables. All extraction and spectrophotometric measurements were performed under reduced light to minimize pigment degradation.

### 4.5. Determination of Osmotic-Adjustment Metabolites

#### 4.5.1. Soluble Sugars

Soluble sugar content was determined using the anthrone–sulfuric acid method as described by Yemm and Willis, with minor modifications [52]. Fresh tissue powder weighing 0.20 g was extracted twice with 5 mL of 80% ethanol at 80 °C for 30 min. After centrifugation at 10,000× *g* for 10 min, the supernatants were combined and adjusted to a known volume with 80% ethanol.

An aliquot of 0.20 mL of extract was mixed with 3.0 mL of freshly prepared anthrone reagent containing 0.2% anthrone in concentrated sulfuric acid. The mixture was heated in a boiling-water bath for 10 min and rapidly cooled on ice. Absorbance was measured at 620 nm. Soluble sugar content was calculated from a glucose standard curve and expressed as mg glucose equivalents g^−1^ fresh weight.

#### 4.5.2. Proline

Free proline was determined using the acid-ninhydrin method described by Bates et al., with minor modifications [53]. Fresh tissue weighing 0.50 g was homogenized in 10 mL of 3% sulfosalicylic acid and centrifuged at 10,000× *g* for 10 min. Two milliliters of supernatant were mixed with 2 mL of acid-ninhydrin reagent and 2 mL of glacial acetic acid. The reaction mixture was incubated at 100 °C for 60 min and rapidly cooled on ice.

The chromophore was extracted with 4 mL of toluene. After phase separation, absorbance of the upper organic phase was measured at 520 nm against a toluene blank. Proline content was calculated from an L-proline standard curve and expressed as µg g^−1^ fresh weight.

#### 4.5.3. Soluble Proteins

Soluble protein content was determined using the Coomassie Brilliant Blue G-250 dye-binding method described by Bradford, with minor modifications [54]. Fresh tissue weighing 0.20 g was homogenized in 2.0 mL of 50 mmol L^−1^ potassium phosphate buffer at pH 7.0 and centrifuged at 12,000× *g* for 15 min at 4 °C. An aliquot of the supernatant was mixed with Coomassie Brilliant Blue G-250 reagent, and absorbance was measured at 595 nm after 5 min. Soluble protein content was calculated from a bovine serum albumin standard curve and expressed as mg g^−1^ fresh weight.

#### 4.5.4. Total Free Amino Acids

Total free amino acids were quantified using the ninhydrin method described by Yemm and Cocking, with minor modifications [55]. Fresh tissue weighing 0.20 g was extracted with 5 mL of 80% ethanol at 80 °C for 30 min and centrifuged at 10,000× *g* for 10 min. One milliliter of extract was mixed with 1 mL of citrate buffer at pH 5.0 and 1 mL of ninhydrin reagent. The mixture was heated at 100 °C for 15 min, cooled to room temperature, and diluted to a fixed volume with 50% ethanol.

Absorbance was measured at 570 nm. Total free amino-acid content was calculated from an L-leucine standard curve and expressed as µmol leucine equivalents g^−1^ fresh weight.

### 4.6. Determination of Oxidative-Stress Indicators

#### 4.6.1. Superoxide Anion

O_2_^−^ content was determined using the hydroxylamine oxidation method described by Elstner and Heupel, with minor modifications [56]. Fresh tissue weighing 0.50 g was homogenized in 5 mL of 65 mmol L^−1^ potassium phosphate buffer at pH 7.8 and centrifuged at 10,000× *g* for 15 min at 4 °C. One milliliter of supernatant was mixed with 0.9 mL of phosphate buffer and 0.1 mL of 10 mmol L^−1^ hydroxylamine hydrochloride and incubated at 25 °C for 20 min.

One milliliter of 17 mmol L^−1^ sulfanilamide and 1 mL of 7 mmol L^−1^ α-naphthylamine were subsequently added. After incubation at 25 °C for a further 20 min, absorbance was measured at 530 nm. Superoxide-dependent nitrite formation was quantified from a sodium nitrite standard curve, and O_2_^−^ content was expressed as nmol g^−1^ fresh weight.

#### 4.6.2. Hydrogen Peroxide

H_2_O_2_ content was determined using the potassium iodide method described by Velikova et al., with minor modifications [57]. Fresh tissue weighing 0.50 g was homogenized in 5 mL of ice-cold 0.1% trichloroacetic acid and centrifuged at 12,000× *g* for 15 min at 4 °C. An aliquot of 0.50 mL of supernatant was mixed with 0.50 mL of 100 mmol L^−1^ potassium phosphate buffer at pH 7.0 and 1.0 mL of 1 mol L^−1^ potassium iodide.

The reaction mixture was incubated in darkness for 60 min, and absorbance was measured at 390 nm. H_2_O_2_ content was calculated from its standard curve and expressed as µmol g^−1^ fresh weight.

#### 4.6.3. Malondialdehyde

MDA concentration was quantified using a commercial 96-well plant-specific double-antibody sandwich enzyme-linked immunosorbent assay (ELISA) kit supplied by Shanghai Enzyme-linked Biotechnology Co., Ltd. (mlBio, Shanghai, China), following the manufacturer’s protocol and standard ELISA principles [58]. The analytical range of the assay was 0.2–4.8 nmol L^−1^.

Ten microliters of tissue extract were mixed with 40 µL of sample diluent in MDA-antibody-coated wells, resulting in a fivefold initial dilution. Fifty microliters of each MDA standard were added to the corresponding standard wells. The plate was incubated at 37 °C for 30 min and washed five times. Fifty microliters of horseradish-peroxidase-labeled detection reagent were subsequently added, followed by a second 30-min incubation at 37 °C and five washing cycles. Color was developed by adding 50 µL each of chromogenic reagents A and B and incubating the plate at 37 °C in darkness for 10 min. The reaction was terminated with 50 µL of stop solution, and absorbance was measured at 450 nm within 15 min.

MDA concentration was calculated from the corresponding standard curve and corrected for the fivefold initial dilution and any additional dilution. Samples exceeding the upper calibration limit were further diluted and reassayed. Results were expressed as nmol L^−1^ of tissue extract.

### 4.7. Determination of Antioxidant Enzyme Protein Levels

SOD, CAT, peroxidase, and GSH-PX were quantified as immunoreactive protein concentrations using commercial plant-specific double-antibody sandwich ELISA kits supplied by Shanghai Enzyme-linked Biotechnology Co., Ltd. (mlBio, Shanghai, China). The analytical ranges were 1–35 pg mL^−1^ for SOD, 1.5–60 ng L^−1^ for CAT, 1–50 ng L^−1^ for POD, and 9.5–350 ng L^−1^ for GSH-PX.

For each analyte, 10 µL of root or shoot extract was mixed with 40 µL of the corresponding sample diluent in analyte-specific antibody-coated wells. Standards were added at 50 µL per well. Plates were incubated at 37 °C for 30 min and washed five times. Fifty microliters of the corresponding horseradish-peroxidase-labeled detection reagent were added, and the plates were incubated at 37 °C for a further 30 min. After five additional washing cycles, 50 µL each of chromogenic reagents A and B were added, and color was developed at 37 °C in darkness for 10 min. The reactions were terminated with 50 µL of stop solution, and absorbance was measured at 450 nm within 15 min.

SOD, CAT, POD, and GSH-PX concentrations were calculated from their respective standard curves and corrected for the fivefold initial dilution and any additional dilution. Samples exceeding the analytical range were further diluted and reassayed. Because these assays quantified immunoreactive protein abundance rather than catalytic reaction rates, the results were reported as protein concentrations or levels rather than enzyme activities.

### 4.8. Determination of AsA–GSH-Cycle Components

#### 4.8.1. Ascorbate and Glutathione Pools

Ascorbate, dehydroascorbate, GSH, and GSSG were quantified using commercial plant-specific double-antibody sandwich ELISA kits supplied by Shanghai Enzyme-linked Biotechnology Co., Ltd. (mlBio, Shanghai, China). The analytical ranges were 10–350 µg L^−1^ for AsA, 12–500 ng L^−1^ for DHA, 3.5–120 ng L^−1^ for GSH, and 2–48 ng L^−1^ for GSSG.

Ten microliters of root or shoot extract were combined with 40 µL of the corresponding sample diluent in analyte-specific antibody-coated wells. Fifty microliters of each standard were added to the standard wells. Plates were incubated at 37 °C for 30 min and washed five times. Fifty microliters of horseradish-peroxidase-labeled detection reagent were then added, followed by a second 30-min incubation at 37 °C. After five additional washing cycles, 50 µL each of chromogenic reagents A and B were added, and color was developed at 37 °C in darkness for 10 min. The reactions were stopped with 50 µL of stop solution, and absorbance was measured at 450 nm within 15 min.

AsA, DHA, GSH, and GSSG concentrations were calculated from the respective standard curves and corrected for the fivefold initial dilution and any additional dilution. Samples above the corresponding analytical ranges were further diluted and reassayed.

#### 4.8.2. AsA–GSH-Cycle Protein Concentrations

APX, monodehydroascorbate reductase, dehydroascorbate reductase, and GR were quantified as immunoreactive protein concentrations using commercial plant-specific double-antibody sandwich ELISA kits supplied by Shanghai Enzyme-linked Biotechnology Co., Ltd. (mlBio, Shanghai, China). The analytical ranges were 1–50 ng L^−1^ for APX, 2–85 ng L^−1^ for MDHAR, 3–120 pg mL^−1^ for DHAR, and 6–200 ng L^−1^ for GR.

Ten microliters of root or shoot extract were mixed with 40 µL of sample diluent in the corresponding antibody-coated wells. Standards were loaded at 50 µL per well. Plates were incubated at 37 °C for 30 min and washed five times. Fifty microliters of the corresponding horseradish-peroxidase-labeled detection reagent were added, followed by a second 30-min incubation at 37 °C. After five washing cycles, 50 µL each of chromogenic reagents A and B were added, and the color was developed at 37 °C in the dark for 10 min. The reactions were stopped with 50 µL of stop solution, and absorbance was measured at 450 nm within 15 min.

APX, MDHAR, DHAR, and GR concentrations were determined from their respective standard curves and corrected for the fivefold initial dilution and any additional dilution. Values exceeding the calibration range were remeasured after appropriate dilution. Because the assays measured the abundance of immunoreactive protein, these results represent protein concentrations rather than catalytic enzyme activities.

### 4.9. Determination of Phytohormones

#### 4.9.1. Stress-Related Phytohormones

Abscisic acid (ABA) concentration was determined using a plant-specific competitive ELISA kit supplied by Reed Biotech Ltd. (Wuhan, China; Plant Abscisic Acid ELISA Kit, catalog no. RE10300). The analytical range was 0.2–16.2 ng mL^−1^, with a stated sensitivity of 0.09 ng mL^−1^. Frozen root and shoot tissues were homogenized separately in ice-cold phosphate-buffered saline (PBS; pH 7.4) and centrifuged at 12,000× *g* for 15 min at 4 °C. The resulting supernatants were diluted when necessary to place the analyte concentration within the corresponding calibration range. Standards and tissue extracts were added to microplate wells pre-coated with ABA and incubated with the corresponding biotinylated antibody. After addition of avidin–horseradish POD conjugate and the required incubation and washing steps, color was developed using tetramethylbenzidine. Absorbance was measured at 450 nm, and ABA concentration was calculated from the corresponding competitive standard curve. Each biological replicate was analyzed in duplicate.

Jasmonic acid (JA) and salicylic acid (SA) were quantified using plant-specific double-antibody sandwich ELISA kits according to the original kit protocols. The nominal analytical ranges were 30–1000 pmol L^−1^ for JA and 50–1500 pmol L^−1^ for SA. The standard series used in the assays were 62.5, 125, 250, 500, and 1000 pmol L^−1^ for JA and 75, 150, 300, 600, and 1200 pmol L^−1^ for SA. The original manuals confirm these analytical ranges and standard concentrations.

For each JA and SA assay, 10 µL of root or shoot tissue extract was mixed with 40 µL of sample diluent in the corresponding antibody-coated wells, resulting in a fivefold initial dilution. Standards were added at 50 µL per well. Plates were incubated at 37 °C for 30 min and washed five times. Fifty microliters of horseradish-peroxidase-labeled detection reagent were then added, followed by a second 30-min incubation at 37 °C. After five additional washing cycles, 50 µL each of chromogenic reagents A and B were added, and color was developed at 37 °C in darkness for 10 min. Reactions were terminated with 50 µL of stop solution, and absorbance was measured at 450 nm within 15 min. The original protocols specify the same fivefold initial dilution procedure for both assays.

JA and SA concentrations were interpolated from their respective standard curves and subsequently multiplied by the fivefold initial dilution factor, together with any additional dilution factor where applicable, to obtain the concentrations in the original tissue extracts. The original protocols explicitly require multiplication of the standard-curve concentration by the sample-dilution factor when calculating the final sample concentration. All sample concentrations interpolated directly from the JA and SA standard curves were within the corresponding calibration ranges. Therefore, the concentrations reported in the Results represent dilution-corrected concentrations of the original tissue extracts rather than the concentrations directly present in the assay wells.

Strigolactone (SL) was quantified using a Plant Strigolactone ELISA Kit (Shanghai Renjie Biological Technology Co., Ltd., Shanghai, China; catalog no. RJ21771) according to the lot-specific manufacturer’s protocol. Absorbance was measured at 450 nm, and SL concentration was calculated from the corresponding standard curve. Because the immunoassay may recognize structurally related strigolactone compounds, the results were reported as immunoreactive strigolactone equivalents.

#### 4.9.2. Growth-Related Phytohormones

Indole-3-acetic acid (IAA) concentration was determined using a plant-specific competitive-inhibition ELISA kit supplied by ELK (Wuhan) Biotechnology Co., Ltd. (Wuhan, China; Plant Indole-3-Acetic Acid ELISA Kit, catalog no. ELK8753). The analytical range was 3.13–200 ng mL^−1^, with a stated sensitivity of 0.88 ng mL^−1^. Frozen root and shoot tissues were homogenized separately in ice-cold PBS (pH 7.4) and centrifuged at 12,000× *g* for 15 min at 4 °C. The resulting supernatants were appropriately diluted, and standards and samples were added to hormone-coated microplate wells and incubated with the corresponding biotinylated detection antibody. Avidin–horseradish POD conjugate was subsequently added, followed by tetramethylbenzidine color development. The reaction was terminated using the supplied stop solution, and absorbance was measured at 450 ± 10 nm. IAA concentrations were determined from the corresponding competitive standard curve and corrected for the applied sample-dilution factors. Each biological replicate was analyzed in duplicate.

Cytokinin (CTK) and gibberellic acid (GA) were quantified using plant-specific double-antibody sandwich ELISA kits according to the original kit protocols. The nominal analytical ranges were 1–48 µg L^−1^ for CTK and 20–480 pg mL^−1^ for GA. The standard series used in the assays were 3, 6, 12, 24, and 48 µg L^−1^ for CTK and 30, 60, 120, 240, and 480 pg mL^−1^ for GA. These ranges and standard concentrations are those specified in the original assay documentation.

For both assays, 10 µL of root or shoot tissue extract was mixed with 40 µL of sample diluent in the corresponding antibody-coated wells, resulting in a fivefold initial dilution. Standards were added at 50 µL per well. Plates were incubated at 37 °C for 30 min and washed five times. Fifty microliters of horseradish-peroxidase-labeled detection reagent were subsequently added, followed by a second 30-min incubation at 37 °C. After five additional washing cycles, 50 µL each of chromogenic reagents A and B were added, and color was developed at 37 °C in darkness for 10 min. Reactions were terminated with 50 µL of stop solution, and absorbance was measured at 450 nm within 15 min. The original CTK and GA protocols specify this fivefold initial dilution procedure.

CTK and GA concentrations were interpolated from their respective standard curves and subsequently multiplied by the fivefold initial dilution factor, together with any additional dilution factor where applicable, to obtain the concentrations in the original tissue extracts. The kit protocols explicitly specify multiplication by the dilution factor when calculating the actual sample concentration. All sample concentrations interpolated directly from the CTK and GA standard curves were within the corresponding calibration ranges. Accordingly, the concentrations reported in the Results represent back-calculated concentrations of the original tissue extracts after correction for the fivefold initial dilution.

### 4.10. Determination of Triacylglycerol Content

Total lipids were extracted from 0.20 g of frozen tissue using chloroform–methanol extraction based on the method of Folch et al., with minor modifications [59]. After vigorous mixing, 0.8 mL of 0.9% NaCl was added to induce phase separation. Samples were centrifuged at 3000× *g* for 10 min, and the lower organic phase was collected. The extraction was repeated, and the combined organic fractions were evaporated under nitrogen.

The dried lipid extract was redissolved in isopropanol. Triacylglycerols were quantified using an enzymatic colorimetric principle based on triacylglycerol hydrolysis to glycerol and fatty acids, followed by coupled glycerol oxidation and chromogenic detection [60]. The released glycerol was phosphorylated by glycerol kinase and subsequently oxidized by glycerol-phosphate oxidase. The resulting H_2_O_2_ was quantified using a peroxidase-coupled chromogenic reaction, and absorbance was measured at 500–510 nm.

A parallel free-glycerol blank was included. Triacylglycerol-associated glycerol was calculated by subtracting free glycerol from total glycerol. TAG content was determined from a triolein or glycerol standard curve and expressed as µmol g^−1^ fresh weight.

### 4.11. Statistical Analysis

The independently irrigated plot was considered the experimental unit. Each treatment comprised three independent plot-level biological replicates, and results are presented as mean ± standard error. Root and shoot tissues were analyzed separately.

Differences among the freshwater control, 8 g L^−1^, and 12 g L^−1^ treatments were evaluated using one-way analysis of variance followed by Tukey’s honestly significant difference test. Normality of residuals and homogeneity of variances were evaluated using the Shapiro–Wilk and Levene tests, respectively. When the assumption of variance homogeneity was not satisfied, Welch’s analysis of variance followed by the Games–Howell post hoc test was applied.

Principal component analysis was conducted separately for root and shoot tissues using z-score-standardized variables and the correlation matrix. Pearson correlation coefficients were calculated from the individual plot-level biological replicates. To account for multiple pairwise comparisons, correlation *p* values were adjusted using the Benjamini–Hochberg false-discovery-rate procedure. Statistical analyses were performed using OriginPro 2024 software (OriginLab Corporation, Northampton, MA, USA). Treatment differences were considered statistically significant at *p* < 0.05, and correlations were considered significant at a false-discovery-rate-adjusted *p* < 0.05.

## 5. Conclusions

Long-term saline irrigation substantially altered the physiological and biochemical status of field-established *N. tangutorum* in the Dananhu coal-mining restoration area. Increasing irrigation-water salinity reduced chlorophyll content, enhanced osmolyte accumulation, increased ROS and MDA levels, and shifted the AsA–GSH system toward a more oxidized state. The 8 g L^−1^ treatment induced stress-associated biochemical adjustment, including higher soluble sugars, proline, soluble proteins, and transient increases in GS and GOGAT, but these responses occurred together with measurable oxidative injury. Under 12 g L^−1^, stronger chlorophyll loss, ROS accumulation, redox oxidation, altered nitrogen-metabolism protein abundance, and stress-hormone accumulation indicated broader physiological disruption.

Overall, the results show that *N. tangutorum* responds to long-term mixed-salt irrigation through coordinated osmotic, redox, nitrogen-metabolic, hormonal, and lipid-related adjustments, but these responses become increasingly constrained at higher salinity. The measured traits provide useful candidate indicators for evaluating downstream physiological responses to saline irrigation. However, because ion homeostasis, plant water status, growth, survival, gas exchange, and recovery were not measured, the present results should not be used to define a salinity-tolerance threshold. Future studies should combine these physiological indicators with plant performance and soil-salinity dynamics to evaluate the restoration suitability of *N. tangutorum* under saline irrigation.

## Figures and Tables

**Table 1:** Effects of salinity treatments on total chlorophyll and carotenoid contents in the shoots of *N. tangutorum*.

Treatment	Total Chlorophyll (mg g^−1^ FW)	Carotenoids (µg g^−1^ FW)
Control	3.92 ± 0.04 ^a^	54.46 ± 1.76 ^a^
8 g L^−1^	3.36 ± 0.15 ^b^	58.87 ± 9.82 ^a^
12 g L^−1^	3.04 ± 0.11 ^c^	67.46 ± 6.46 ^a^

Note: Values are presented as the mean ± SE of three independent biological replicates (n = 3). Different lowercase letters within the same column indicate significant differences among salinity treatments according to Tukey’s honestly significant difference test at *p* < 0.05.

## Data Availability

The data supporting the findings of this study are available from the corresponding author upon reasonable request.

## Supplementary Materials

The following supporting information can be downloaded at: https://www.mdpi.com/article/10.3390/ijms27177694/s1.
