# Supplementary material for: Physiological and Biochemical Responses of Nitraria tangutorum to Long-Term Saline Irrigation in an Arid Coal-Mining Restoration Area
Source: Int J Mol Sci. 2026 Aug 28;27(17):7694. doi: 10.3390/ijms27177694 (PMC13565838; doi:10.3390/ijms27177694)
Supplement: Supplementary file 1 [file ijms-27-07694-s001.zip › ijms-4471955-supplementary.pdf]

Table S1. Commercial ELISA kits and analytical characteristics used in this study

**Analyte**	**Supplier**	**Catalogue/lot information**	**Analytical range**	**Initial dilution**	**Wavelength**	**Reporting unit**
MDA	Shanghai Enzyme-linked Biotechnology Co., Ltd. (mlBio), Shanghai, China	Not retained in archived documentation	0.2–4.8 nmol L^{-1}	5-fold	450 nm	nmol L^{-1}
SOD	Same supplier	Not retained in archived documentation	1–35 pg mL^{-1}	5-fold	450 nm	pg mL^{-1}
CAT	Same supplier	Not retained in archived documentation	1.5–60 ng L^{-1}	5-fold	450 nm	ng L^{-1}
POD	Same supplier	Not retained in archived documentation	1–50 ng L^{-1}	5-fold	450 nm	ng L^{-1}
GSH-PX	Same supplier	Not retained in archived documentation	9.5–350 ng L^{-1}	5-fold	450 nm	ng L^{-1}
AsA	Same supplier	Not retained in archived documentation	10–350 µg L^{-1}	5-fold	450 nm	µg L^{-1}
DHA	Same supplier	Not retained in archived documentation	12–500 ng L^{-1}	5-fold	450 nm	ng L^{-1}
GSH	Same supplier	Not retained in archived documentation	3.5–120 ng L^{-1}	5-fold	450 nm	ng L^{-1}
GSSG	Same supplier	Not retained in archived documentation	2–48 ng L^{-1}	5-fold	450 nm	ng L^{-1}

**Analyte**	**Supplier**	**Catalogue/lot information**	**Analytical range**	**Initial dilution**	**Wavelength**	**Reporting unit**
APX	Same supplier	Not retained in archived documentation	1–50 ng L^{-1}	5-fold	450 nm	ng L^{-1}
MDHAR	Same supplier	Not retained in archived documentation	2–85 ng L^{-1}	5-fold	450 nm	ng L^{-1}
DHAR	Same supplier	Not retained in archived documentation	3–120 pg mL^{-1}	5-fold	450 nm	pg mL^{-1}
GR	Same supplier	Not retained in archived documentation	6–200 ng L^{-1}	5-fold	450 nm	ng L^{-1}
NR	Same supplier	Not retained in archived documentation	1–50 ng L^{-1}	5-fold	450 nm	ng L^{-1}
GS	Same supplier	Not retained in archived documentation	2–80 ng L^{-1}	5-fold	450 nm	ng L^{-1}
GOGAT	Same supplier	Not retained in archived documentation	6–200 pg mL^{-1}	5-fold	450 nm	pg mL^{-1}

Table S2. Commercial ELISA kits used for the determination of phytohormones.

Analyte	Manufacturer	Location	Catalogue no.	Assay principle	Nominal analytical range	Standard series used	Sensitivity	Initial sample dilution	Sample type	Detection wavelength
ABA	Reed Biotech Ltd.	Wuhan, China	RE10300	Competitive ELISA	0.2–16.2 ng mL^{-1}	According to manufacturer's protocol	0.09 ng mL^{-1}	As required	Plant-tissue extract	450 nm
JA	Shanghai Renjie Biological Technology Co., Ltd.	Shanghai, China	RE10303	Double-antibody sandwich ELISA	30–1000 pmol L^{-1}	62.5, 125, 250, 500, 1000 pmol L^{-1}	Not stated	5-fold	Plant-tissue extract	450 nm
SA	Shanghai Renjie Biological Technology Co., Ltd.	Shanghai, China	RE10301	Double-antibody sandwich ELISA	50–1500 pmol L^{-1}	75, 150, 300, 600, 1200 pmol L^{-1}	Not stated	5-fold	Plant-tissue extract	450 nm
SL	Shanghai Renjie Biological Technology Co., Ltd.	Shanghai, China	RJ21771	Plant SL ELISA	Lot-specific	Lot-specific	Lot-specific	According to lot-specific protocol	Plant-tissue extract	450 nm
IAA	ELK (Wuhan) Biotechnology Co., Ltd.	Wuhan, China	ELK8753	Competitive-inhibition ELISA	3.13–200 ng mL^{-1}	According to manufacturer's protocol	0.88 ng mL^{-1}	As required	Plant-tissue extract	450 ± 10 nm
CTK	Shanghai Renjie Biological Technology Co., Ltd.	Shanghai, China	ELK8628	Double-antibody sandwich ELISA	1–48 µg L^{-1}	3, 6, 12, 24, 48 µg L^{-1}	Not stated	5-fold	Plant-tissue extract	450 nm
GA	Shanghai Renjie Biological Technology Co., Ltd.	Shanghai, China	RE10302	Double-antibody sandwich ELISA	20–480 pg mL^{-1}	30, 60, 120, 240, 480 pg mL^{-1}	Not stated	5-fold	Plant-tissue extract	450 nm

Notes: Analytical ranges and standard series for CTK, GA, SA, and JA were verified against the original kit instruction sheets and assay records. For these four assays, 10 µL of tissue extract was mixed with 40 µL of sample diluent, resulting in a fivefold initial dilution. Concentrations interpolated from the corresponding standard curves were multiplied by the fivefold dilution factor to obtain the concentrations in the original tissue extracts. All sample concentrations interpolated directly from the standard curves were within their respective calibration ranges before application of the dilution correction. Reported concentrations therefore represent dilution-corrected concentrations of the original tissue extracts.
